# Historical Redlining Is Associated with Disparities in Environmental Quality across California

**DOI:** 10.1021/acs.estlett.3c00870

**Published:** 2024-01-19

**Authors:** Cesar O. Estien, Christine E. Wilkinson, Rachel Morello-Frosch, Christopher J. Schell

**Affiliations:** † Department of Environmental Science, Policy, and Management, University of California−Berkeley, 130 Mulford Hall, Berkeley, California 94720, United States; ‡ California Academy of Sciences, 55 Music Concourse Drive, San Francisco, California 94118, United States; § School of Public Health, University of California−Berkeley, 2121 Berkeley Way, Berkeley, California 94720, United States

**Keywords:** environmental justice, pollution, noise, inequity, redlining, CalEnviroScreen

## Abstract

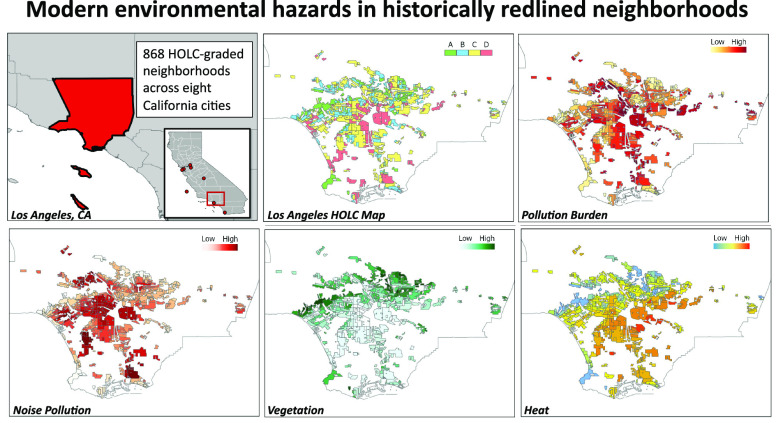

Historical policies have been shown to underpin environmental quality. In the 1930s, the federal Home Owners’ Loan Corporation (HOLC) developed the most comprehensive archive of neighborhoods that would have been redlined by local lenders and the Federal Housing Administration, often applying racist criteria. Our study explored how redlining is associated with environmental quality across eight California cities. We integrated HOLC’s graded maps [grades A (i.e., “best” and “greenlined”), B, C, and D (i.e., “hazardous” and “redlined”)] with 10 environmental hazards using data from 2018 to 2021 to quantify the spatial overlap among redlined neighborhoods and environmental hazards. We found that formerly redlined neighborhoods have poorer environmental quality relative to those of other HOLC grades via higher pollution, more noise, less vegetation, and elevated temperatures. Additionally, we found that intraurban disparities were consistently worse for formerly redlined neighborhoods across environmental hazards, with redlined neighborhoods having higher pollution burdens (77% of redlined neighborhoods vs 18% of greenlined neighborhoods), more noise (72% vs 18%), less vegetation (86% vs 12%), and elevated temperature (72% vs 20%), than their respective city’s average. Our findings highlight that redlining, a policy abolished in 1968, remains an environmental justice concern by shaping the environmental quality of Californian urban neighborhoods.

## Introduction

Urban environmental quality varies significantly due to differences in access to wealth and resources,^1,2^ implicating societal inequities in perpetuating disparities in environmental quality. Heterogeneity in urban environmental quality and wealth is largely driven by racial segregation, racialized zoning practices, and other forms of racist government actions (e.g., limiting civic engagement of marginalized populations).^3−5^ These dynamics have resulted in communities of color being disproportionately exposed to environmental hazards.^6,7^ It is therefore critical to unpack how specific policies have perpetuated environmental harm in order to develop restorative policies.

Racial segregation is a practice and societal structure that underlies the spatial distribution of environmental hazards (e.g., heat risk,^8^ noise^9^). A notable process formalizing racial segregation in the United States was redlining, a policy instituted by the Home Owners’ Loan Corporation (hereafter HOLC) following the Great Depression.^10,11^ In the process, the HOLC created risk assessment maps to identify neighborhoods that would have been redlined by local lenders and the Federal Housing Administration via a ranking system denoting neighborhood quality,^12^ from best (i.e., grade A, or “greenlined”) to hazardous (i.e., grade D, or “redlined”). However, their determination of decline, hazards, and investment risk were directly linked to Black and immigrant communities, with appraisers often noting the ethno-racial composition of neighborhoods.^10−13^ The process of redlining reflected and codified existing racist practices, including zoning laws and discriminatory housing practices, leading to continued disinvestment in redlined neighborhoods.^11,12^

Formerly redlined neighborhoods have distinct ecologies compared to greenlined neighborhoods.^2,14^ Recent research has shown that previously redlined neighborhoods have, for example, poorer air quality and intensified urban heat islands.^15−18^ Due to these environmental inequities, humans residing in redlined neighborhoods today demonstrate higher rates of adverse health outcomes, including cancer,^19^ cardiovascular disease,^20^ and asthma.^21^ Such outcomes may not be restricted to humans, as emerging research suggests wildlife species are also being impacted,^2,22,23^ with potential additional negative feedbacks that affect human health.^24,25^ Thus, understanding the association between redlining and environmental quality is crucial for mitigating and deconstructing the potential consequences for human and wildlife health within cities.

While prior redlining studies typically focus on a single metric, such as canopy cover,^26^ hazards often co-occur spatially. Understanding where environmental hazards co-occur is crucial for assessing the potential synergistic interactions between them and the consequential cumulative impact on human and nonhuman organisms. Yet, comprehensive work examining multiple hazards across multiple cities is scarce, despite the high applicability for informing state and federal environmental justice policies.

Here, we leveraged HOLC maps to examine whether the practice of redlining is associated with environmental quality in California cities. We focused on California as it is the most populous state in the US (∼39 million people^27^), with 43 cities ranking in the top 200 largest US cities by population.^28^ To assess environmental quality, we extracted relevant variables from CalEnviroScreen, a high-resolution environmental hazard mapping tool that uses the most recent publicly available data.^29^ Notably, while CalEnviroScreen’s story map examines the relationship between redlining and environmental hazards,^30^ it lacks a formal analysis that controls for city-level differences and neighborhood size. Thus, we evaluate environmental quality by examining the spatial distribution of various environmental hazards using CalEnviroScreen4.0 alongside other data sources (see Methods and Materials). We hypothesized that previously redlined neighborhoods would have poorer environmental quality (i.e., higher pollution, more noise, less vegetation, and elevated temperatures) than nonredlined neighborhoods.

## Methods and Materials

### Data Sets and Geospatial Processing

We obtained HOLC-graded maps for Fresno, Los Angeles, Oakland, Sacramento, San Diego, San Francisco, San Jose, and Stockton via the Mapping Inequality project^13^ (S1.1). Across California, there are 868 HOLC-graded neighborhoods: 109 A-graded, 273 B-graded, 331 C-graded, and 155 D-graded. We evaluated the following environmental hazards to assess environmental quality in relation to redlining: groundwater threats, lead risk from housing, particulate matter 2.5 (PM_2.5_), diesel particulate matter, toxic releases from facilities, hazardous waste generators and facilities, cleanup sites (i.e., brownfield sites), normalized difference vegetation index (NDVI), temperature, and noise pollution (S1.2). Geospatial analyses were conducted using ArcGIS Pro utilizing the “Zonal Statistics” tool to extract the mean for all hazards. Statistical analyses were completed in R v.4.1.0.^31^

We extracted the mean CalEnviroScreen4.0 score for each hazard per neighborhood and converted each score to a percentile to assess disparities between HOLC grades. We scaled it such that a score of 1 represents no environmental hazard burden, and a score of 100 represents the highest burden. We then used these percentiles, following CalEnviroScreen methods,^29^ to produce a pollution burden for each neighborhood. The pollution burden metric represents a cumulative score of multiple environmental hazards within a neighborhood and includes groundwater threats, lead risk from housing, PM_2.5_, diesel PM, toxic releases from facilities, hazardous waste generators and facilities, and cleanup sites (S1.2).

To calculate NDVI and temperature, we used Landsat 8 OLI Level 1 (C2 L1) terrain-correct, with images from December 2020 and January 2021 where cloud cover was <20% and retained the appropriate bands (S1.2). We used these bands to calculate NDVI, which represents the amount of vegetation with lower values corresponding with less vegetation and land surface temperature in degree Celsius. To calculate noise pollution, we used HowLoud (https://howloud.com), which calculates noise pollution values caused by local traffic, airplane traffic, and other local sources of noise (S1.2).

### Data Analysis

We ran general-linear mixed models to understand the effect of HOLC grades on environmental quality with HOLC grade as a fixed effect and city as a random effect to control for potential among-city differences using the *glmmTMB* package^32^ (SM 1.3). We also included the area of a neighborhood as a log-offset variable to control for the fact that larger neighborhoods, by virtue of size, may have higher environmental hazards. We performed Tukey–Kramer’s posthoc analyses to determine which HOLC grade dyads (e.g., A vs C, A vs D) differed in the focal environmental hazard (S1.3). We report the mean, standard deviation, and D-grade comparisons below for each environmental hazard. We report model results and all pairwise comparisons in Supporting Information S2 and S3.

To further understand disparities in environmental quality, we considered intraurban disparities via investigating the relative difference per environmental hazard at the city-level between a neighborhood and their respective city. We did this by calculating a city’s average for each hazard, then subtracted a neighborhood’s environmental quality estimate from the city’s average,^13^ such that a value of 0 would represent no disparity between a neighborhood’s environmental hazard and the corresponding city’s average value for that hazard. We then compared the interquartile range (IQR) (ANOVA comparisons are shown in the Supporting Information).

## Results

### Environmental Quality

**Figure 1:**
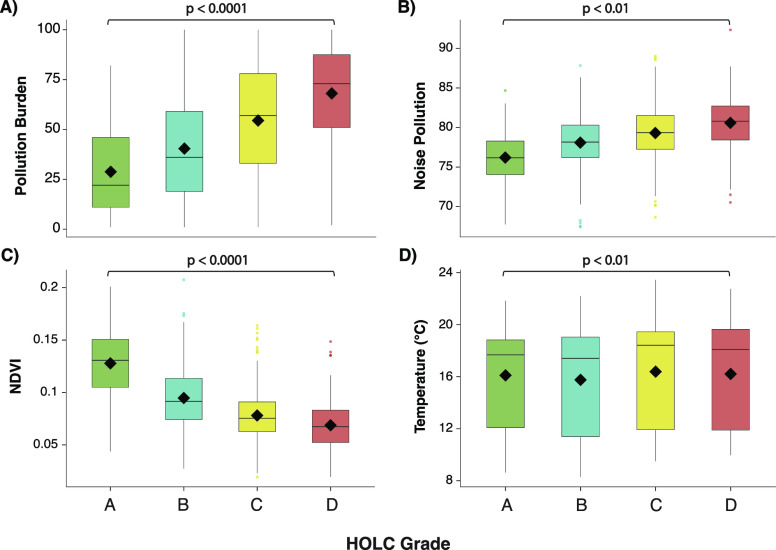
Redlined neighborhoods have higher environmental hazards than nonredlined neighborhoods. We show (A) pollution burden, (B) noise pollution, (C) vegetation (NDVI*), and (D) temperature across HOLC grades. Pollution burden is a score based on the presence of various environmental hazard (see Methods and Materials). Noise pollution is on a scale of 50–100, with 100 representing a very loud environment. NDVI (i.e., vegetation) is on a scale of −1 to 1, with lower scores representing less vegetation. Black diamonds represent the mean and whiskers represent 95% confidence intervals. *NDVI = Normalized Differentiated Vegetation Index.

After controlling for the area of a neighborhood and among-city variation, we found a strong relationship between HOLC grade and environmental quality (Figure 1; Table 1; S2.1; Figure S1). Across all environmental hazards, redlined neighborhoods had higher pollution burdens, less vegetation, more noise pollution, and higher temperatures (Figure 1; Table 1; Figure S1). Overall, HOLC grades significantly predict overall pollution burden for a neighborhood, with redlined neighborhoods having a significantly higher pollution burden than nonredlined neighborhoods (Table 1; Figure 1). Though redlined neighborhoods had higher pollution burdens than greenlined neighborhoods in every city but one (Figure S2), we only found significant differences in pollution burden between greenlined and redlined neighborhoods in five of the eight cities (Table S2). We found similar variation for each environmental hazard. Redlined and nonredlined neighborhoods showed no significant differences in PM_2.5_ and toxic releases, but did show significant differences in lead risk, groundwater threats, hazardous waste facilities, cleanup sites, and diesel PM (Table 1), with variation at the city-level for each environmental hazard (Figures S3–S7; Table S2). For example, although we found no significant differences overall in PM_2.5_ and toxic releases, we found significant differences in these hazards in three and four cities, respectively (Figures S8, S9; Table S2). We found significant differences between redlined and nonredlined neighborhoods in noise pollution, NDVI, and temperature (Figure 1B–D; Table 1). Noise pollution, NDVI, and temperature varied significantly in seven of the eight cities (Figures S10–S12; Table S2), though differences were not always between redlined and nonredlined neighborhoods.

**Table 1:** Environmental Hazards in California by HOLC grade

Environmental Hazard	Grade A(*n* = 109)	Grade B(*n* = 273)	Grade C(*n* = 331)	Grade D(*n* = 155)	A–D p-value	B–D p-value	C–D p-value
PM_2.5_	45.9 (23.8)	45.2 (27.5)	51.2 (29.3)	54.4 (31.0)	*p* = 0.6867	*p* = 0.6105	*p* = 0.7534
Diesel PM	29.5 (23.9)	41.3 (26.8)	53.3 (26.6)	68.5 (24.9)	*p* < 0.0001	*p* < 0.001	*p* < 0.01
Lead risk	35.5 (23.4)	46.9 (27.2)	52.3 (29.0)	56.5 (29.8)	*p* = 0.4856	*p* < 0.05	*p* = 0.9681
Groundwater threat	35.4 (24.9)	45.5 (27.8)	52.1 (28.3)	59.4 (28.3)	*p* < 0.01	*p* = 0.7998	*p* = 0.0936
Toxic releases by facilities	46.8 (25.6)	47.0 (27.2)	50.2 (30.2)	53.0 (29.3)	*p* = 0.7855	*p* = 0.0813	*p* = 0.8140
Hazardous waste facilities	36.0 (22.8)	44.2 (27.7)	52.4 (27.7)	60.7 (30.3)	*p* < 0.001	*p* = 0.4328	*p* < 0.05
Cleanup sites	39.0 (26.5)	43.5 (27.9)	51.1 (26.9)	62.5 (29.6)	*p* < 0.0001	*p* < 0.01	*p* < 0.0001
Pollution burden	28.8 (21.8)	40.4 (25.9)	54.5 (27.8)	68.1 (24.1)	*p* < 0.0001	*p* = 0.0001	*p* < 0.0001
Noise pollution	76.2 (3.5)	78.1 (3.2)	79.3 (3.2)	80.6 (3.4)	*p* < 0.01	*p* < 0.0001	*p* < 0.01
NDVI	0.13 (0.03)	0.09 (0.03)	0.08 (0.02)	0.07 (0.02)	*p* < 0.0001	*p* < 0.0001	*p* < 0.001
Temperature (°C)	16.1 (3.7)	15.8 (3.8)	16.4 (3.7)	16.2 (3.9)	*p* < 0.01	*p* < 0.0001	*p* < 0.001

Raw data for each environmental hazard is shown as mean (standard deviation) across HOLC grades (grades A = “best” and “greenlined”; B, C, and D = “hazardous” and “redlined”). PM_2.5_, diesel particulate matter, lead risk from housing, groundwater threat, toxic releases from facilities, hazardous waste facilities, cleanup sites, and pollution burden are shown as percentiles (1–100), where 1 represents no environmental hazard burden, and 100 represents a high burden. Noise pollution is on a scale of 50–100, with 100 representing a very loud environment using HowLoud’s soundscore (https://howloud.com). NDVI (i.e., vegetation) is on a scale of −1 to 1, with lower scores representing less vegetation. The number of graded neighborhoods for each HOLC grade is shown above the respective column. Pair-wise comparison between greenlined and redlined neighborhoods from generalized linear mixed-models that control for the area of a neighborhood and among-city variation are shown. Significant comparisons from Tukey–Kramer’s posthoc analyses are bolded. The remaining pairwise comparisons are found in Table S1 in Supporting Information.

### Intraurban Disparities

**Figure 2:**
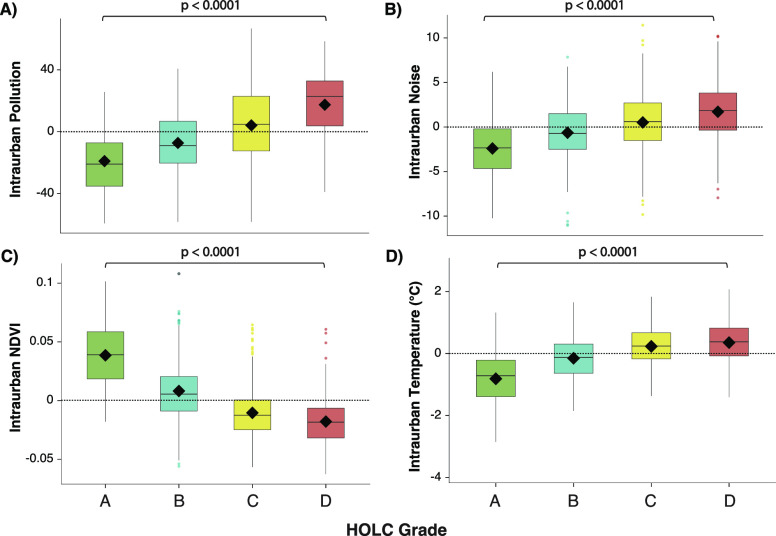
Redlined neighborhoods disproportionately face worse intraurban disparities in environmental hazards than nonredlined neighborhoods. Intraurban disparity analysis for (A) overall pollution burden, (B) noise pollution, (C) vegetation (NDVI*), and (D) temperature across HOLC grades. Horizontal zero line represents no difference between the city’s average and a neighborhood’s average environmental hazard. For graphs A–D, values above the line represent a higher environmental hazard value (i.e., higher pollution, more noise, and elevated temperature) than the corresponding cities average. For graph C, values below the line represent less vegetation value than the corresponding cities average. Black diamonds represent the mean and whiskers represent 95% confidence intervals. *NDVI = Normalized Differentiated Vegetation Index.

Grade D ubiquitously exhibited environmental hazards that were worse than the city’s average (Figure 2; S2.2; Tables S3), with strong significance found between redlined and nonredlined neighborhoods (Table S4). Pollution was higher than average in 77% of redlined neighborhoods compared to 18% of greenlined neighborhoods across all cities (Figure 2A), with no IQR overlap for seven cities (i.e., A-graded 75th percentile was lower than D-graded 25th percentile; Figure S13). Similar directionality was observed for lead (61% of redlined vs 29% of greenlined neighborhoods) as well as water contamination (70% of redlined vs 29% of greenlined neighborhoods) (Figure S14), with both hazards showing no IQR overlap for three cities (Figures S15, S16). For air pollutants (i.e., PM_2.5_, diesel PM, and toxic releases), we found the same directionality again (Figure S14). PM_2.5_ was higher than average in 70% of redlined neighborhoods compared to 31% greenlined neighborhoods, with no IQR overlap for three cities (Figures S14, S17). Diesel PM was higher than average in 75% of redlined neighborhoods compared to 24% greenlined neighborhoods, with no IQR overlap for six cities (Figures S14, S18). Similarly, toxic releases were higher than average in 61% of redlined neighborhoods compared to 35% greenlined neighborhoods, with no IQR overlap for three cities (Figures S14, S19). We found the same directionality for cleanup sites (66% of redlined vs 43% of greenlined neighborhoods) and hazardous waste facilities (64% of redlined vs 30% of greenlined neighborhoods) (Figure S14), with both hazards showing no IQR overlap for two cities (Figures S20, S21). For noise pollution, 72% of redlined neighborhoods had higher levels of noise than average compared to 18% of greenlined neighborhoods (Figure 2B), with no IQR overlap for seven cities (Figure S22). For NDVI, 86% of redlined neighborhoods had less than average vegetation compared to 12% of greenlined neighborhoods with no IQR overlap for all cities (Figure 2C; Figure S23). Similar disparities were observed for temperature, with 72% of redlined neighborhoods having higher temperatures than average compared to 20% of greenlined neighborhoods (Figure 2D). For temperature, there was no IQR overlap for the six cities (Figure S24).

## Discussion

### Redlining and Environmental Quality

Redlining persisted across the United States from 1933 to 1968 in over 230 cities.^10,11^ Alongside restrictive deeds, racialized zoning, steering, and social violence, redlining led to land use and decision making that worsened the environmental quality for redlined neighborhoods.^2,10,14^ In this study, we assessed 868 previously HOLC-graded neighborhoods across eight California cities and found that redlining is strongly associated with environmental quality. Our results enrich the redlining literature by demonstrating that formerly redlined neighborhoods exhibit significantly poorer environmental quality than other HOLC grades, including less investigated hazards such as noise pollution, lead, and contaminated water.^33−35^ This holds implications for human health disparities, as redlined neighborhoods in California are composed of census tracts with higher proportions of Hispanic and Black populations, as well as a people living in poverty, than greenlined neighborhoods.^36^ Our results shed light on the enduring, far-reaching impact of redlining, a policy that was abolished almost 60 years ago, on contemporary environmental quality.

We found that redlined neighborhoods consistently face disproportionately worse environmental hazards with respect to their city’s average (i.e., intraurban disparity) across all 10 assessed hazards, often by several orders of magnitude. Intraurban analysis revealed that, despite some hazards not showing differences in environmental quality between HOLC grades, at either the state or city level, intraurban disparities are still strong. For example, though we did not find significant differences between greenlined and redlined neighborhoods for PM_2.5_ across all cities, we found large intraurban disparities, with redlined neighborhoods having double the proportion of neighborhoods that face higher levels of PM_2.5_ than greenlined neighborhoods. Thus, intraurban analyses, which explicitly examine relative city-level differences in environmental quality, may better capture contemporary social factors (e.g., contemporary lines of segregation) than general state-level analyses, despite controlling for city-level differences.

### City-Level Variation in Environmental Hazards

The emergent patterns between redlining and environmental quality may vary by the city for several reasons. Since HOLC maps were drawn in the 1930s and 1940s, urban expansion has dramatically increased,^37^ resulting in many cities expanding beyond the boundaries of their original HOLC maps. For instance, much of the Oakland metropolitan area and San Francisco lie within their original HOLC maps, compared to Fresno, Stockton, and Sacramento, which have experienced substantial growth, with most of the geographic expands of these cities now outside their respective HOLC geographic boundaries. Due to this sprawl and the associated movements in sociodemographics, more contemporary factors may be overriding the legacy effects produced by redlining. Namely, gentrification in these cities may ameliorate prior pollution burdens, resulting in concerted reinvestment that neutralizes differences among HOLC grades. Indeed, Californian cities are rapidly gentrifying,^38^ with an influx of wealth and development potentially improving green space availability, quality, and distribution.^39^ Modifications to the green infrastructure of certain neighborhoods can subsequently reduce urban heat, help to purify the air, and buffer against urban noise.^40,41^

Variation in environmental hazards may be influenced by a multitude of social and ecological factors. Notably, the unique geography and urban layouts of each California city, such as the distance to open water, highway concentration, population density, and housing distribution, may directly impact environmental hazard burdens. San Francisco, a coastal city on a peninsula, for example, has a 27% PM_2.5_ burden, whereas Fresno, a city surrounded by mountains, has a 97% PM_2.5_ burden. Yet, Los Angeles, another costal city, faces a PM_2.5_ burden (71%) similar to that of Fresno, despite its coastal location and dense infrastructure. Thus, the geographic and microclimatic conditions of each city may mediate the burdens experienced by human and nonhuman organisms. In parallel, contemporary policies and governance may also have mitigated or eliminated disparities in environmental quality, resulting in the lack of statistical differences across graded neighborhoods. Fresno, for example, where we found no differences in PM_2.5_ between HOLC grades, has undergone several management strategies since 1992 to reduce air pollution in the city and the greater San Joaquin, leading exposure to PM_2.5_ to be reduced by 85%, respectively.^42^

While no differences were found between redlined and nonredlined neighborhoods for certain environmental hazards, disparities may still exist. For instance, within the United States, racial–ethnic disparities for air pollution,^43,44^ chemical toxins,^45^ and water quality^46^ still persist. Within California, recent research has shown that racially marginalized communities throughout California continue to face disproportionate exposure to oil and gas wells and the associated disturbances,^47^ higher levels of water contamination,^48^ and lower reductions in PM_2.5_.^49^ Thus, although redlining is generally understood to underpin environmental quality, our results show that this may vary by city, and leveraging sociodemographic information (e.g., socioeconomics, race) in tandem with HOLC maps may be critical for elucidating environmental quality disparities.

### Implications

Our results show that across California, redlined neighborhoods have disproportionately worse environmental quality than other HOLC grades, further implicating redlining as a major driver of contemporary disparities in environmental quality. These environmental inequities are multigenerational and will stubbornly persist without proper intervention and remediation.^25^ We hope results from this work are pertinent to decision makers at the city- and state-levels by pinpointing neighborhoods that disproportionately suffer heavy environmental quality burdens. Moreover, this work holds potential for urban One Health initiatives, which recognize the shared health and well-being of the environment, people, and wildlife, highlighting that ongoing efforts to enhance urban resilience can benefit from considering legacy effects. Our work emphasizes that potential interventions for environmental inequities via policy, such as the White House’s Justice40 initiative,^50^ must center social justice to effectively address environmental injustices produced by systemic racism.

## Supporting Information Available

Detailed description of methods and materials, as well as Tables S1–S4 and Figures S1–S24, including city specific results (PDF)
