# Supplementary material for: Historical Redlining Is Associated with Disparities in Environmental Quality across California
Source: Environ Sci Technol Lett. 2024 Jan 19;11(2):54–9. doi: 10.1021/acs.estlett.3c00870 (PMC10867848; doi:10.1021/acs.estlett.3c00870)
Supplement: Supplementary file 1 — ez3c00870_si_001.pdf [file ez3c00870_si_001.pdf]

Supporting Information

Historical redlining is associated with disparities in environmental quality across California

Cesar O. Estien*[1], Christine E. Wilkinson[1,2], Rachel Morello-Frosch[1,3], and Christopher J. Schell[1]

Affiliations:
[1]Department of Environmental Science, Policy, and Management, 130 Mulford Hall, University of California–Berkeley, Berkeley, CA 94720, USA
[2]California Academy of Sciences, 55 Music Concourse Drive, San Francisco, CA 94118, USA
[3]School of Public Health, 2121 Berkeley Way, University of California–Berkeley, Berkeley, CA 94720, USA

*Corresponding author: Cesar O. Estien, cestien@berkeley.edu

Contents:
Pages S1-S37
Table S1-S4
Figures S1-S24

S1: Material and Methods

S1.1: Study region

Our study takes place throughout California. We focused on California because of accessibility to the high-resolution environmental hazard mapping tool CalEnviroscreen 4.0[1]. Within California, eight cities have digitized Home Owner's Loan Corporation (HOLC) maps via the University of Richmond's Mapping Inequality project[2]: Fresno, Los Angeles, Oakland, Sacramento, San Diego, San Francisco, San Jose, and Stockton. Note, the Oakland HOLC map includes Oakland, Berkeley, San Leandro, Piedmont, Emeryville, and Albany, and the Los Angeles map includes the greater Los Angeles area[2].

S1.2: Datasets and Geospatial Processing

CalEnviroScreen uses an array of measures (e.g., groundwater threats) and air quality (e.g., ozone) to produce a cumulative pollution burden percentile for each census tract[1]. We followed CalEnviroScreen methodology to produce our own pollution burden for a neighborhood based on the hazards we downloaded. We first converted each environmental hazard into a raster before extracting the mean for an environmental hazard per HOLC neighborhood. We extracted children's lead risk from housing, particulate matter 2.5 ($PM_{2.5}$), diesel particulate matter, toxic releases from facilities, groundwater threats, which is based on the activates that can pose a threat to groundwater quality (e.g., land disposal sites, underground storage tanks, and animal farms), hazardous waste generators and facilities, which represents sites permitted to treat, store, or dispose of hazardous waste, and cleanup sites (i.e., brownfield sites). Read more about how CalEnviroScreen derives each hazardous metric at https://oehha.ca.gov/calenviroscreen.

To create a pollution burden for each neighborhood, we averaged what CalEnviroScreen considers an exposure (lead risk, $PM_{2.5}$, diesel PM, toxic releases from facilities) and an environmental effect (groundwater threats, hazardous waste generators and facilities, cleanup sites) into an "exposure" and "effect" variable. We then combine the exposure and effect variables, with the effect score only weighed half as much as the exposure[1] (see Eq. 1-2). CalEnviroScreen does this as the contribution to the pollution burden a neighborhood experience comes less from hazards in the "effects" category and more from the "exposure" category. The "effects" category simply reflects the presence of these environmental hazards rather than direct exposure to them. After combining environmental hazards and averaging them (Eq. 3), we then binned them into percentiles such that pollution burden would be on a scale of 1 to 100, such that a score of 1 represents no environmental hazard burden and a score of 100 represents the highest burden.

$$EXPOSURE = \frac{\text{LEAD} + \text{PM2.5} + \text{DIESEL PM} + \text{TOXIC RELEASES}}{4} \qquad \textbf{Eq. 1}$$

$$EFFECT = \frac{\text{GROUNDWATER} + \text{HAZARD RELEASES} + \text{CLEANUP}}{3} \qquad \textbf{Eq. 2}$$

$$Pollution\ Burden = \frac{(EXPOSURE + (EFFECT * 0.5))}{(1 + 0.5)} \qquad \textbf{Eq. 3}$$

For Landsat 8 satellite imagery, we selected the year 2020 and 2021 to best align with the most recent data layers of CalEnviroScreen (see paragraph below), and we selected December and

January because we wanted to understand disparities in vegetation during the wetter part of the year (i.e., highest vegetation). We downloaded Bands 4 and 5 to calculate Normalized Differentiated Vegetation Index (NDVI) (see Equation 4) and Band 10 to calculate land surface temperature (Equations 5-6).

$$NDVI = \frac{NIR - Red}{NIR + Red} \quad \textbf{Eq. 4}$$

$$NIR = Near\ Infrared\ Band$$

To calculate land surface temperature, we first calculated the top of atmospheric (TOA) spectral radiance using the following equation from:

$$TOA = M_L * Q_{cal} + A_L \quad \textbf{Eq. 5}$$

$$M_L = Band - specific\ multiplicative\ rescaling\ factor$$
$$Q_{cal} = Band$$
$$A_L = Band - specific\ additive\ rescaling\ factor$$

After calculating the TOA, we then converted the values into Celsius to obtain land-surface temperature with the following equation:

$$T = \left(\frac{K_1}{(\ln\left(\frac{K_2}{L}\right) + 1))}\right) - 273.15 \quad \textbf{Eq. 6}$$

$$K_1 = Band - specific\ thermal\ conversion\ constant\ from\ the\ metadata$$
$$K_2 = Band - specific\ thermal\ conversion\ constant\ from\ the\ metadata$$

To calculate noise pollution, we extracted data from HowLoud.com, which scales noise pollution from 50, representing high levels of noise, and 100, representing high levels of silence. For each city, we extracted values for 2,000 random points within that city's HOLC map. After obtaining these values, we rasterized each point dataset using the 'Kriging' function. In short, the kriging function interpolates data to infer values for particular spaces between points where sampling did not occur[3].After extracting data from HowLoud, we inverted the scale for visualization purposes but retained the original values for use in our models (see S1.3)

S1.3: Data Analysis

To understand the influence of HOLC grade on the spatial distribution of environmental hazards, we ran generalized linear mixed models (GLMMs) with HOLC grade as the fixed effect, the area of a neighborhood as a log-offset variable, and city as a random effect using the *glmmTMB* package[4]. We repeated this model approach at the city-level, but removed city as a random effect and used a general linear model with the *betareg*[5]. For all environmental hazards except NDVI, temperature, and noise we used a beta distribution given the data were bounded between 0 and 1. For NDVI, temperature, and noise, we used a log-linked gaussian distribution. We built two models for environmental hazards: a model containing HOLC grade as an independent variable and a null model where HOLC grade was omitted.

$$HOLC\ Model: Environmental\ Hazard \sim HOLC\ Grade + offset\ (Neighborhood\ Area) + (1 \mid city)$$

$$Null\ Model: Environmental\ Hazard \sim 1\ +\ offset\ (Neighborhood\ Area) + (1 \mid city)$$

We then used an AICc model-selection approach, selecting the models with the lowest AICc value. When the top-performing model was identified, we tested for significant differences between the top-performing model and the null model using likelihood ratio tests (LRT). If the differences were significant, we extracted the estimated marginal means and performed Tukey-Kramer's post-hoc analyses to determine which specific HOLC grade dyads (e.g., A vs. C, A vs. D, etc.) differed in the focal environmental hazard(s). Model selection results are found in Supporting Information 2.

References

1. California Office of Environmental Health Hazard Assessment. CalEnviroScreen 4.0. https://oehha.ca.gov/calenviroscreen/report/draft-calenviroscreen-40 (accessed 2023-11-29).
2. Nelson, R. K., Winling, L., Marciano, R., Connolly, N. & Ayers, E. L. Mapping inequality: Redlining in new deal America. *American Panorama: An Atlas of United States History. University of Richmond: Digital Scholarship Lab* **17**, 19 (2020).
3. OLIVER, M. A. & WEBSTER, R. Kriging: a method of interpolation for geographical information systems. *International journal of geographical information systems* **4**, 313–332 (1990).
4. Magnusson, A. *et al.* Package 'glmmtmb'. *R Package Version 0.2. 0* **25**, (2017).
5. Zeileis, A. *et al.* Package 'betareg'. *R package* **3**, (2016).

S2: Supporting Information Results

S2.1: Model Results

We found a strong relationship between HOLC grade and environmental quality (Figure 1; Table 1; SM 2). We found a significant effect of HOLC grade on a neighborhoods pollution burden (Likelihood Ratio Test (LRT) = 71.3; $p < 0.0001$), particulate matter 2.5 (hereafter $PM_{2.5}$) (LRT = 9.1; $p < 0.05$) diesel pm (hereafter diesel PM) (LRT = 54.5; $p < 0.0001$), lead risk (LRT = 23.7; $p < 0.0001$), groundwater threat (LRT = 15.9; $p < 0.01$), toxic releases by facilities (LRT = 18.3; $p = 0.0004$), hazardous waste facilities (LRT = 17.7; $p = 0.0005$), cleanup sites (LRT = 37.7; $p < 0.0001$), amount of vegetation (LRT = 101.1; $p < 0.0001$), temperature (LRT = 101.1; $p < 0.0001$), and noise (LRT = 85.57; $p < 0.0001$). We found variation for the effect of HOLC grade on environmental hazards at the city-level (Table S2; Figures S2-10).

S2.2: Intraurban Disparities

We found significant differences in intraurban disparities between HOLC grades across the environmental hazards examined. For pollution burden, grade D held the highest disparity (17.4 ± 20.9; Table S3), followed by grades C, B, and A and we found significant differences between all pairwise comparisons (Table S4). For lead, grade D held the highest disparity (5.4 ± 28.4; Table S3), followed by grades C, B, and A. We found significant differences between all pairwise comparisons except B and C and C and D (Table S4). For groundwater threat, grade D held the highest disparity (9.5 ± 24.0; Table S3), followed by grades C, B, and A. We found significant differences between grades A and B as well as A and D (Table S4). For toxic releases, grade D held the highest disparity (3.7 ± 13.8; Table S3), followed by grades C, B, and A and we found significant differences between all grades except A and B as well as B and C (Table S4). For hazardous waste facilities, grade D held the highest disparity (11.3 ± 29.8; Table S3), followed by grades C, B, and A and we found significant differences between all grades except A and B as well as B and C (Table S4). For cleanup sites, grade D held the highest disparity (13.0 ± 28.6; Table S3), followed by grades C, B, and A and we found significant differences between all grades except A and B as well as B and C (Table S4). For diesel PM, grade D held the highest disparity (18.4 ± 23.8; Table S3), followed by grades C, B, and A, with all pair-wise comparisons showing significant differences (Table S4). For $PM_{2.5}$, grade D held the highest disparity (4.2 ± 12.3; Table S3), followed by grades C, B, and A, with all pair-wise comparisons showing significant differences (Table S4). For NDVI, grade D had the lowest disparity (-0.02 ± 0.02; Table S3), followed by grades C, B, and A. We found significant differences between all pairwise comparisons for NDVI (Table S4). For temperature, grade D had the highest disparity (0.4 ± 0.8; Table S3), followed by grades C, B, and A. We found significant differences in thermal intensity between all pairwise comparisons except grades C and D (Table S4). Lastly, for noise pollution, grade D had the highest disparity (1.7 ± 3.2; Table S3), followed by grades C, B, and A. We found significant differences between all pairwise comparisons for noise pollution (Table S4).

S3: Supporting Information Tables

Environmental Hazard	A-B	A-C	A-D	B-C	B-D	C-D
*$PM_{2.5}$	$p = 0.1069$	$p = 0.9765$	$p = 0.6867$	**$p < 0.05$**	$p = 0.6105$	$p = 0.7534$
Diesel PM	**$p < 0.0001$**	**$p < 0.0001$**	**$p < 0.0001$**	$p = 1$	**$p < 0.001$**	**$p < 0.01$**
Lead Risk	**$p = 0.0001$**	$p = 0.1646$	$p = 0.4856$	**$p < 0.01$**	**$p < 0.05$**	$p = 0.9681$
Groundwater threat	**$p < 0.01$**	$p = 0.1597$	**$p < 0.01$**	$p = 0.3309$	$p = 0.7998$	$p = 0.0936$
Toxic Releases by Facilities	**$p < 0.05$**	$p = 0.9905$	$p = 0.7855$	**$p < 0.001$**	$p = 0.0813$	0.8140
Hazardous Waste Facilities	**$p < 0.05$**	$p = 0.3537$	**$p < 0.001$**	$p = 0.2913$	$p = 0.4328$	**$p < 0.05$**
Cleanup Sites	**$p < 0.05$**	$p = 0.3493$	**$p < 0.0001$**	$p = 0.1795$	**$p < 0.01$**	**$p < 0.0001$**
Pollution Burden	**$p < 0.0001$**	**$p < 0.0001$**	**$p < 0.0001$**	$p = 0.9999$	**$p = 0.0001$**	**$p < 0.0001$**
NDVI	**$p < 0.001$**	**$p < 0.0001$**	**$p < 0.0001$**	**$p < 0.0001$**	**$p < 0.0001$**	**$p < 0.001$**
Heat	**$p < 0.0001$**	$p = 0.9898$	**$p < 0.01$**	**$p < 0.0001$**	**$p < 0.0001$**	**$p < 0.001$**
Noise Pollution	**$p < 0.0001$**	$p = 0.9882$	**$p < 0.01$**	**$p < 0.0001$**	**$p < 0.0001$**	**$p < 0.01$**

Table S1. Pair-wise comparisons for environmental hazards across California from generalized linear mixed-models after controlling for the area of a neighborhood and among-city variation. We used Tukey-Kramer's post-hoc analyses to determine which specific HOLC grade dyads (e.g., A vs. C, A vs. D, etc.) significantly differed in the focal environmental hazard(s). Significant comparisons are bolded. *$PM_{2.5}$ = particulate matter 2.5

City	Environmental Hazard	A-B	A-C	A-D	B-C	B-D	C-D
Fresno	*$PM_{2.5}$						
Fresno	Diesel PM	$p = 0.9996$	$p = 0.3360$	$p = 0.2404$	**$p = 0.05$**	**$p < 0.05$**	$p = 0.9175$
Fresno	Lead Risk	$p = 0.3380$	$p = 0.5474$	$p = 0.9495$	$p = 0.3259$	**$p < 0.01$**	$p = 0.0608$
Fresno	Groundwater threat						
Fresno	Toxic Releases by Facilities						
Fresno	Hazardous Waste Facilities						
Fresno	Cleanup Sites						
Fresno	Pollution Burden						
Fresno	NDVI	$p = 0.0763$	**$p < 0.001$**	**$p < 0.05$**	$p = 0.0610$	$p = 0.9534$	$p = 0.1694$
Fresno	Temperature (°C)	$p = 0.3156$	**$p < 0.05$**	$p = 0.2698$	$p = 0.1359$	$p = 1$	$p = 0.0702$
Fresno	Noise Pollution	$p = 0.2125$	**$p < 0.01$**	$p = 0.1470$	$p = 0.1104$	$p = 0.9990$	$p = 0.0943$
Los Angeles	$PM_{2.5}$	**$p = 0.0005$**	**$p < 0.0001$**	**$p < 0.0001$**	**$p < 0.001$**	**$p < 0.0001$**	**$p < 0.001$**
Los Angeles	Diesel PM	**$p < 0.0001$**	**$p < 0.0001$**	**$p < 0.0001$**	**$p = 0.0001$**	**$p < 0.0001$**	**$p < 0.0001$**
Los Angeles	Lead Risk	**$p < 0.01$**	**$p < 0.0001$**	**$p < 0.0001$**	**$p < 0.01$**	**$p < 0.0001$**	$p = 0.1679$
Los Angeles	Groundwater threat	**$p = 0.0001$**	**$p < 0.0001$**	**$p < 0.0001$**	$p = 0.7363$	**$p < 0.001$**	**$p < 0.01$**
Los Angeles	Toxic Releases by Facilities	$p = 0.7866$	**$p = 0.0001$**	**$p < 0.0001$**	**$p = 0.0002$**	**$p < 0.0001$**	**$p < 0.0001$**
Los Angeles	Hazardous Waste Facilities	**$p < 0.01$**	**$p < 0.0001$**	**$p < 0.0001$**	$p = 0.4175$	**$p < 0.0001$**	**$p < 0.0001$**
Los Angeles	Cleanup Sites	$p = 0.1135$	**$p = 0.0003$**	**$p < 0.0001$**	$p = 0.2266$	**$p < 0.0001$**	**$p = 0.0004$**
Los Angeles	Pollution Burden	**$p < 0.0001$**	**$p < 0.0001$**	**$p < 0.0001$**	**$p < 0.0001$**	**$p < 0.0001$**	**$p < 0.0001$**
Los Angeles	NDVI	**$p < 0.01$**	**$p < 0.0001$**	**$p < 0.0001$**	**$p < 0.0001$**	**$p < 0.0001$**	$p = 0.2785$
Los Angeles	Temperature (°C)	**$p < 0.0001$**	$p = 0.9766$	$p = 0.4686$	**$p < 0.0001$**	**$p < 0.0001$**	$p = 0.4631$
Los Angeles	Noise Pollution	**$p < 0.0001$**	$p = 0.5903$	$p = 0.0871$	**$p < 0.0001$**	**$p < 0.0001$**	$p = 0.3737$
Oakland	$PM_{2.5}$						
Oakland	Diesel PM	**$p < 0.01$**	**$p < 0.0001$**	**$p < 0.0001$**	$p = 0.1127$	**$p < 0.0001$**	**$p < 0.0001$**
Oakland	Lead Risk	**$p < 0.01$**	**$p < 0.0001$**	**$p < 0.0001$**	$p = 0.1687$	**$p < 0.01$**	$p = 0.5187$
Oakland	Groundwater threat	**$p < 0.001$**	**$p < 0.0001$**	**$p < 0.0001$**	**$p < 0.0001$**	**$p < 0.0001$**	**$p < 0.0001$**

Oakland	Toxic Releases by Facilities	$p = 0.6413$	**$p < 0.05$**	$p = 0.9767$	$p = 0.1851$	$p = 0.8014$	**$p < 0.05$**
Oakland	Hazardous Waste Facilities	$p = 0.7717$	$p = 0.8691$	**$p < 0.0001$**	$p = 0.9932$	**$p < 0.0001$**	**$p < 0.0001$**
Oakland	Cleanup Sites	$p = 0.9996$	$p = 0.8736$	**$p < 0.0001$**	$p = 0.7057$	**$p < 0.0001$**	**$p < 0.0001$**
Oakland	Pollution Burden	**$p < 0.001$**	**$p < 0.0001$**	**$p < 0.0001$**	**$p < 0.05$**	**$p < 0.0001$**	**$p < 0.0001$**
Oakland	NDVI	**$p < 0.001$**	$p = 0.7368$	$p = 0.1089$	**$p < 0.01$**	**$p < 0.0001$**	**$p < 0.05$**
Oakland	Temperature (°C)	**$p < 0.01$**	**$p < 0.05$**	$p = 0.8893$	$p = 0.2965$	**$p = 0.0004$**	**$p < 0.05$**
Oakland	Noise Pollution	**$p < 0.01$**	$p = 0.0772$	$p = 1$	$p = 0.0906$	**$p < 0.0001$**	**$p < 0.05$**
Sacramento	$PM_{2.5}$	$p = 0.9781$	$p = 0.9959$	$p = 0.1721$	$p = 0.7225$	$p = 0.1286$	**$p < 0.05$**
Sacramento	Diesel PM						
Sacramento	Lead Risk						
Sacramento	Groundwater threat						
Sacramento	Toxic Releases by Facilities	$p = 0.9679$	$p = 0.9829$	**$p < 0.05$**	$p = 0.5220$	**$p < 0.05$**	**$p = 0.0001$**
Sacramento	Hazardous Waste Facilities						
Sacramento	Cleanup Sites	$p = 0.3607$	$p = 0.1907$	$p = 0.5458$	$p = 0.9973$	**$p = 0.0001$**	**$p < 0.0001$**
Sacramento	Pollution Burden	$p = 0.8047$	$p = 0.9681$	**$p < 0.05$**	$p = 0.8337$	**$p < 0.05$**	**$p < 0.001$**
Sacramento	NDVI	$p = 0.3663$	$p = 0.9343$	$p = 0.9988$	**$p < 0.001$**	$p = 0.3015$	$p = 0.7847$
Sacramento	Temperature (°C)	$p = 0.3121$	$p = 0.9992$	$p = 0.8371$	**$p < 0.01$**	$p = 0.5311$	$p = 0.5523$
Sacramento	Noise Pollution	$p = 0.3831$	$p = 0.9998$	$p = 0.8544$	**$p < 0.01$**	$p = 0.6537$	$p = 0.4194$
San Diego	$PM_{2.5}$	**$p < 0.05$**	**$p < 0.05$**	**$p < 0.0001$**	$p = 1$	**$p < 0.0001$**	**$p < 0.0001$**
San Diego	Diesel PM	$p = 0.7281$	**$p < 0.05$**	$p = 0.0596$	$p = 0.2731$	$p = 0.3719$	$p = 0.9955$
San Diego	Lead Risk	$p = 0.9988$	$p = 0.9665$	**$p < 0.0001$**	$p = 0.9838$	**$p < 0.0001$**	**$p < 0.0001$**
San Diego	Groundwater threat						
San Diego	Toxic Releases by Facilities	$p = 0.3983$	$p = 0.2369$	**$p < 0.0001$**	$p = 0.9922$	**$p = 0.0001$**	**$p < 0.001$**
San Diego	Hazardous Waste Facilities						
San Diego	Cleanup Sites	$p = 0.9997$	$p = 0.3023$	$p = 0.9981$	$p = 0.1900$	$p = 0.9868$	**$p < 0.05$**
San Diego	Pollution Burden	$p = 0.9992$	$p = 0.9965$	$p = 0.0775$	$p = 0.9997$	**$p < 0.05$**	$p = 0.0568$
San Diego	NDVI	**$p < 0.05$**	**$p < 0.0001$**	**$p < 0.0001$**	**$p < 0.05$**	**$p < 0.0001$**	**$p < 0.01$**
San Diego	Temperature (°C)	$p = 0.6361$	**$p < 0.05$**	**$p < 0.0001$**	$p = 0.1469$	**$p < 0.0001$**	**$p < 0.001$**
San Diego	Noise Pollution	$p = 0.4258$	**$p < 0.01$**	**$p < 0.0001$**	$p = 0.0872$	**$p < 0.0001$**	**$p < 0.001$**
San Francisco	$PM_{2.5}$						
San Francisco	Diesel PM	$p = 0.8836$	**$p < 0.01$**	**$p < 0.0001$**	**$p < 0.0001$**	**$p < 0.0001$**	**$p < 0.0001$**

San Francisco	Lead Risk						
San Francisco	Groundwater threat	$p = 0.5771$	$p = 0.8981$	**$p < 0.0001$**	**$p < 0.05$**	**$p < 0.0001$**	**$p < 0.0001$**
San Francisco	Toxic Releases by Facilities						
San Francisco	Hazardous Waste Facilities	$p = 0.5975$	$p = 0.8859$	$p = 0.0986$	$p = 0.8663$	**$p < 0.0001$**	**$p < 0.0001$**
San Francisco	Cleanup Sites						
San Francisco	Pollution Burden	$p = 0.9957$	$p = 0.9923$	**$p < 0.0001$**	$p = 0.8725$	**$p < 0.0001$**	**$p < 0.0001$**
San Francisco	NDVI	**$p < 0.0001$**	**$p < 0.0001$**	**$p < 0.0001$**	$p = 0.8954$	$p = 0.8396$	$p = 0.4520$
San Francisco	Temperature (°C)	**$p < 0.001$**	**$p < 0.01$**	**$p = 0.0001$**	$p = 0.5308$	$p = 0.9921$	$p = 0.3487$
San Francisco	Noise Pollution	**$p = 0.0001$**	**$p < 0.01$**	**$p < 0.0001$**	$p = 0.6643$	$p = 0.8907$	$p = 0.2668$
San Jose	$PM_{2.5}$						
San Jose	Diesel PM	$p = 0.4617$	$p = 0.9985$	$p = 0.9086$	$p = 0.0682$	**$p < 0.001$**	$p = 0.2357$
San Jose	Lead Risk						
San Jose	Groundwater threat						
San Jose	Toxic Releases by Facilities						
San Jose	Hazardous Waste Facilities						
San Jose	Cleanup Sites						
San Jose	Pollution Burden	$p = 0.5345$	$p = 1$	$p = 0.9557$	**$p < 0.05$**	**$p < 0.01$**	$p = 0.6152$
San Jose	NDVI						
San Jose	Temperature (°C)	$p = 0.8947$	$p = 0.9956$	$p = 0.6401$	$p = 0.3713$	$p = 0.9032$	**$p < 0.05$**
San Jose	Noise Pollution	$p = 0.8781$	$p = 0.9981$	$p = 0.5144$	$p = 0.3874$	$p = 0.7902$	**$p < 0.05$**
Stockton	$PM_{2.5}$	$p = 0.9651$	$p = 0.5506$	**$p < 0.05$**	$p = 0.6140$	**$p < 0.01$**	$p = 0.1055$
Stockton	Diesel PM	$p = 0.9265$	$p = 0.5164$	**$p < 0.05$**	$p = 0.6869$	**$p < 0.01$**	$p = 0.2084$
Stockton	Lead Risk						
Stockton	Groundwater threat	$p = 0.6739$	$p = 0.9463$	$p = 0.9357$	$p = 0.0624$	**$p < 0.05$**	$p = 1$
Stockton	Toxic Releases by Facilities						
Stockton	Hazardous Waste Facilities						
Stockton	Cleanup Sites	$p = 0.6393$	**$p < 0.05$**	**$p < 0.01$**	$p = 0.0533$	**$p < 0.05$**	$p = 1$
Stockton	Pollution Burden	$p = 0.6889$	**$p < 0.05$**	**$p < 0.001$**	**$p < 0.05$**	**$p = 0.0001$**	$p = 0.3316$

Stockton	NDVI	p = 0.9774	**p < 0.05**	**p < 0.01**	p = 0.0533	**p < 0.05**	p = 1
Stockton	Temperature (°C)						
Stockton	Noise Pollution						

Table S2. Pair-wise comparisons for environmental hazards in Californian cities from generalized linear models that control for the area of a neighborhood. Environmental hazards that showed significance was followed by a Tukey-Kramer's post-hoc analyses to determine which specific HOLC grade dyads (e.g., A vs. C, A vs. D, etc.) significantly differed in the focal environmental hazard(s). Significant comparisons for Tukey-Kramer's post-hoc analyses are bolded. Grey rows indicate no significant differences were found between HOLC grades. *$PM_{2.5}$ = particulate matter 2.5

Environmental Hazard	Grade A<br>(n = 109)	Grade B<br>(n = 273)	Grade C<br>(n = 331)	Grade D<br>(n = 155)
$PM_{2.5}$	-5.2 (10.7)	-1.8 (10.9)	1.2 (11.7)	4.2 (12.3)
Diesel PM	-18.5 (22.7)	-7.9 (25.9)	4.0 (26.5)	18.4 (23.8)
Lead Risk	-10.7 (21.0)	-2.0 (23.4)	2.6 (26.7)	5.4 (28.4)
Groundwater threat	-10.5 (22.0)	-3.7 (21.9)	2.1 (22.6)	9.5 (24.0)
Toxic Releases by Facilities	-4.4 (11.5)	-0.9 (11.5)	0.4 (12.1)	3.7 (13.8)
Hazardous Waste Facilities	-2.1 (22.1)	-4.6 (26.9)	2.5 (27.1)	11.3 (29.8)
Cleanup Sites	-8.6 (26.0)	-4.8 (26.2)	0.7 (25.2)	13.0 (28.6)
Pollution Burden	-19.0 (21.2)	-7.3 (20.8)	4.1 (22.7)	17.4 (20.9)
NDVI	0.04 (0.03)	0.01 (0.03)	-0.01 (0.02)	-0.02 (0.02)
Temperature (°C)	-0.8 (1.0)	-0.2 (0.9)	0.2 (0.7)	0.4 (0.8)
Noise Pollution	-2.4 (3.3)	-0.6 (3.2)	0.5 (3.1)	1.7 (3.2)

Table S3. Intraurban disparity data for each environmental hazard shown is as mean (standard deviation) across HOLC grades (grades A = “best” and “greenlined”, B, C, and D = “hazardous” and “redlined”). The number of graded neighborhoods for each HOLC grade is shown above the respective column. *$PM_{2.5}$ = particulate matter 2.5

**Environmental Hazard**	**A-B**	**A-C**	**A-D**	**B-C**	**B-D**	**C-D**
$PM_{2.5}$	**$p < 0.05$**	**$p < 0.0001$**	**$p < 0.0001$**	**$p < 0.01$**	**$p < 0.0001$**	**$p < 0.05$**
Diesel PM	**$p < 0.01$**	**$p < 0.0001$**	**$p < 0.0001$**	**$p < 0.0001$**	**$p < 0.0001$**	**$p < 0.0001$**
Lead Risk	**$p < 0.05$**	**$p < 0.0001$**	**$p < 0.0001$**	$p = 0.1230$	**$p < 0.05$**	$p = 0.6661$
Groundwater threat	**$p < 0.05$**	**$p < 0.0001$**	**$p < 0.0001$**	**$p < 0.05$**	**$p < 0.0001$**	**$p < 0.01$**
Toxic Releases by Facilities	$p = 0.0561$	**$p < 0.01$**	**$p < 0.0001$**	$p = 0.554$	**$p < 0.01$**	**$p < 0.05$**
Hazardous Waste Facilities	$p = 0.0676$	**$p < 0.0001$**	**$p < 0.0001$**	**$p < 0.01$**	**$p < 0.0001$**	**$p < 0.01$**
Cleanup Sites	$p = 0.5801$	**$p < 0.01$**	**$p < 0.0001$**	$p = 0.0504$	**$p < 0.0001$**	**$p < 0.0001$**
Pollution Burden	**$p < 0.0001$**	**$p < 0.0001$**	**$p < 0.0001$**	**$p < 0.0001$**	**$p < 0.0001$**	**$p < 0.0001$**
NDVI	**$p < 0.0001$**	**$p < 0.0001$**	**$p < 0.0001$**	**$p < 0.0001$**	**$p < 0.0001$**	**$p < 0.01$**
Temperature (°C)	**$p < 0.0001$**	**$p < 0.0001$**	**$p < 0.0001$**	**$p < 0.0001$**	**$p < 0.0001$**	$p = 0.40$
Noise Pollution	**$p < 0.0001$**	**$p < 0.0001$**	**$p < 0.0001$**	**$p < 0.0001$**	**$p < 0.0001$**	**$p < 0.001$**

Table S4. Pair-wise comparisons for environmental hazards across California for intraurban disparity data via ANOVA. We used Tukey-Kramer's post-hoc analyses to determine which specific HOLC grade dyads (e.g., A vs. C, A vs. D, etc.) significantly differed in the focal environmental hazard(s). Significant comparisons are bolded. *$PM_{2.5}$ = particulate matter 2.5

S4: Supporting Information Figures

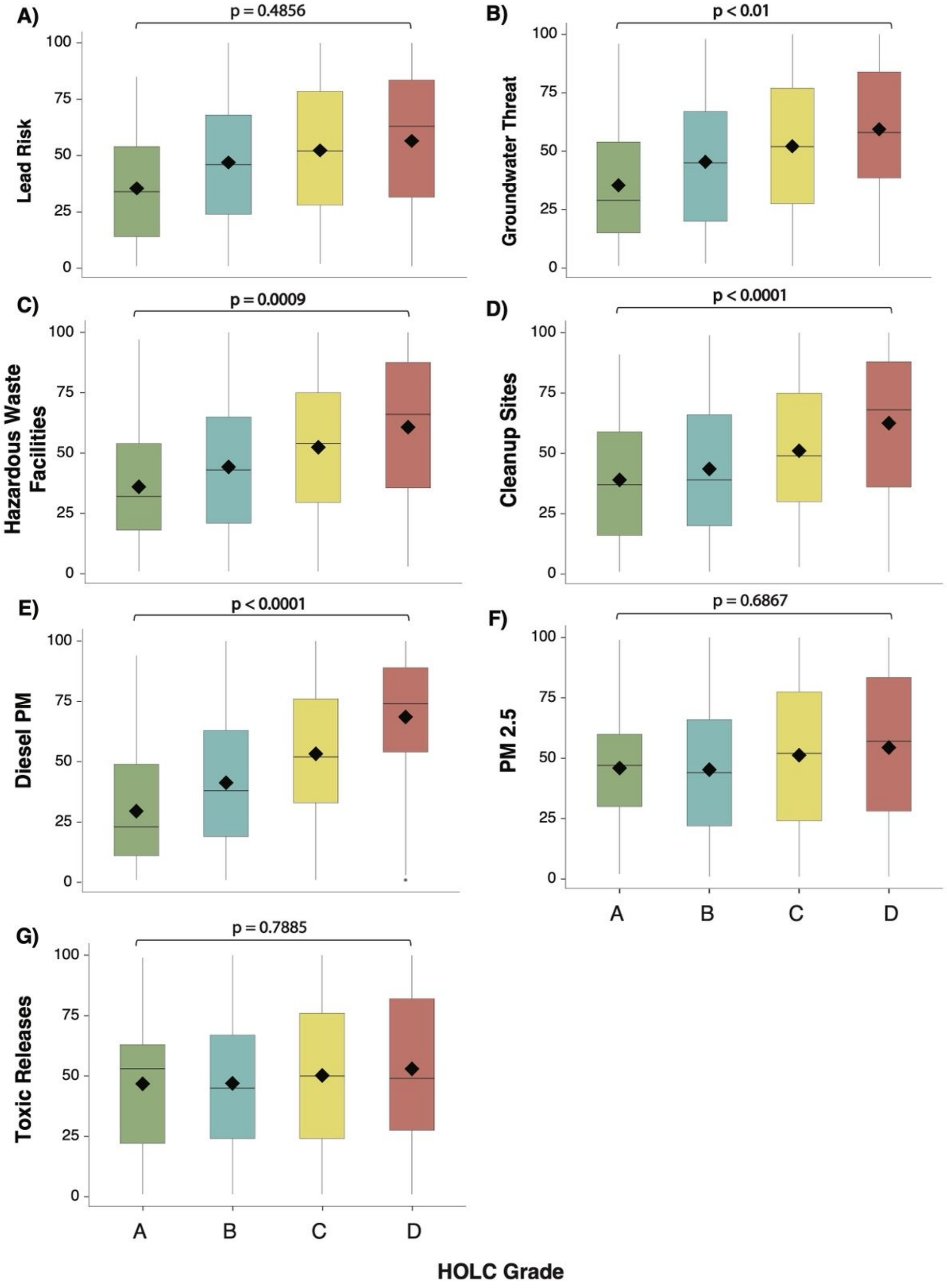

Figure S1. The relationship between HOLC grade and environmental hazards. We show (A) lead risk from housing, (B) water contamination, (C) hazardous waste facilities, (D) cleanup sites, (E) diesel particulate matter, (F) particulate matter (pm) 2.5 and (G) toxic releases from facilities across formerly graded HOLC neighborhoods. Measurements are shown in box plots where each dot represents a measurement within a neighborhood. The mean is shown as a black diamond, and whiskers represent 95% confidence intervals.

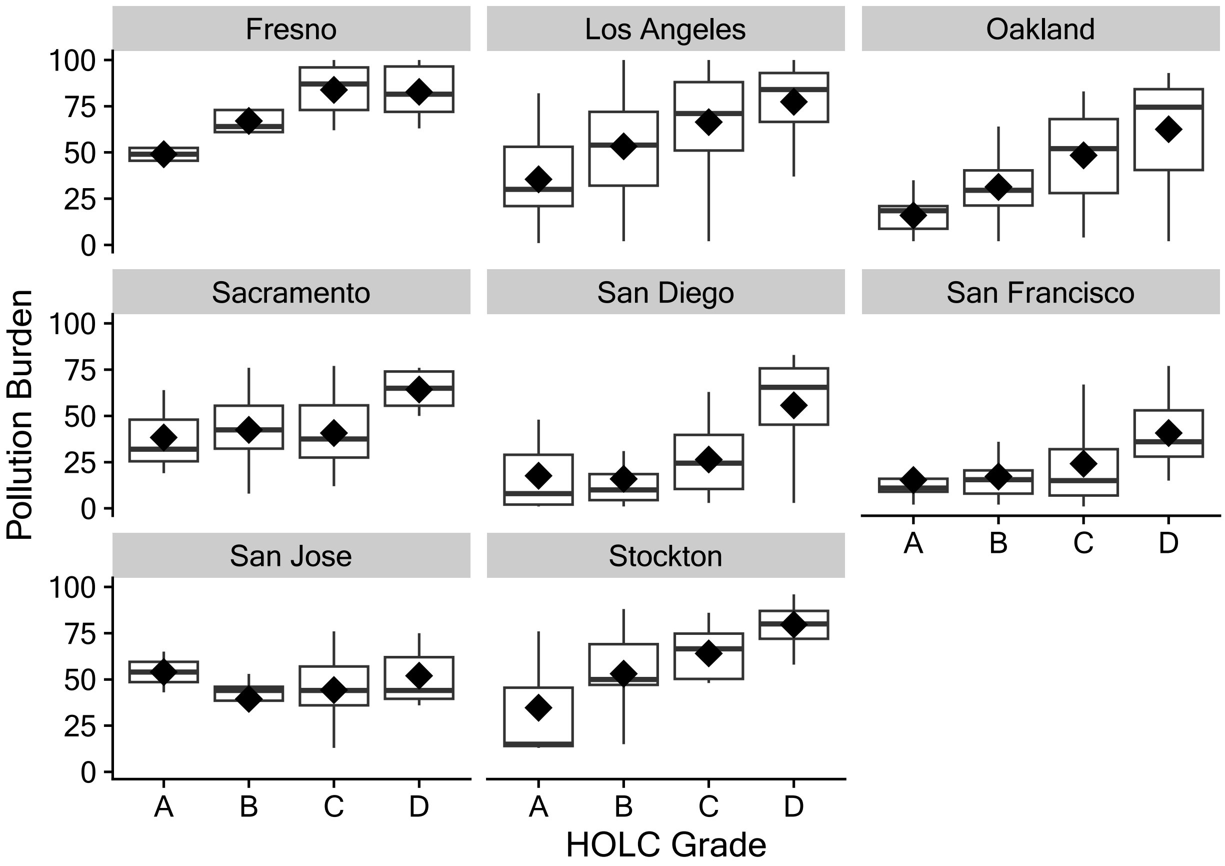

Figure S2. The relationship between HOLC grade and overall pollution burden for each city in our dataset. Measurements are shown in box plots where each dot represents a measurement within a neighborhood. The mean is shown as a black diamond, and whiskers represent 95% confidence intervals.

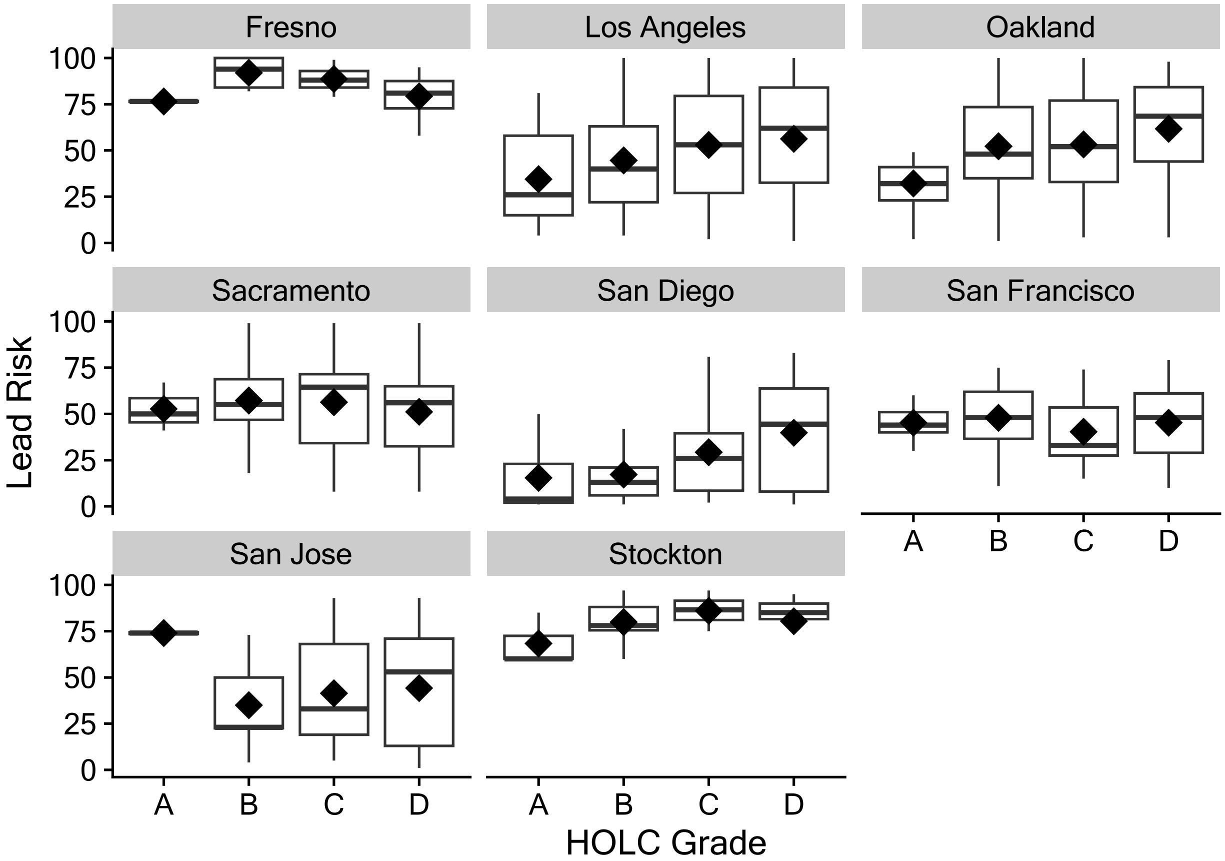

Figure S3. The relationship between HOLC grade and lead risk from housing for each city in our dataset. Measurements are shown in box plots where each dot represents a measurement within a neighborhood. The mean is shown as a black diamond, and whiskers represent 95% confidence intervals.

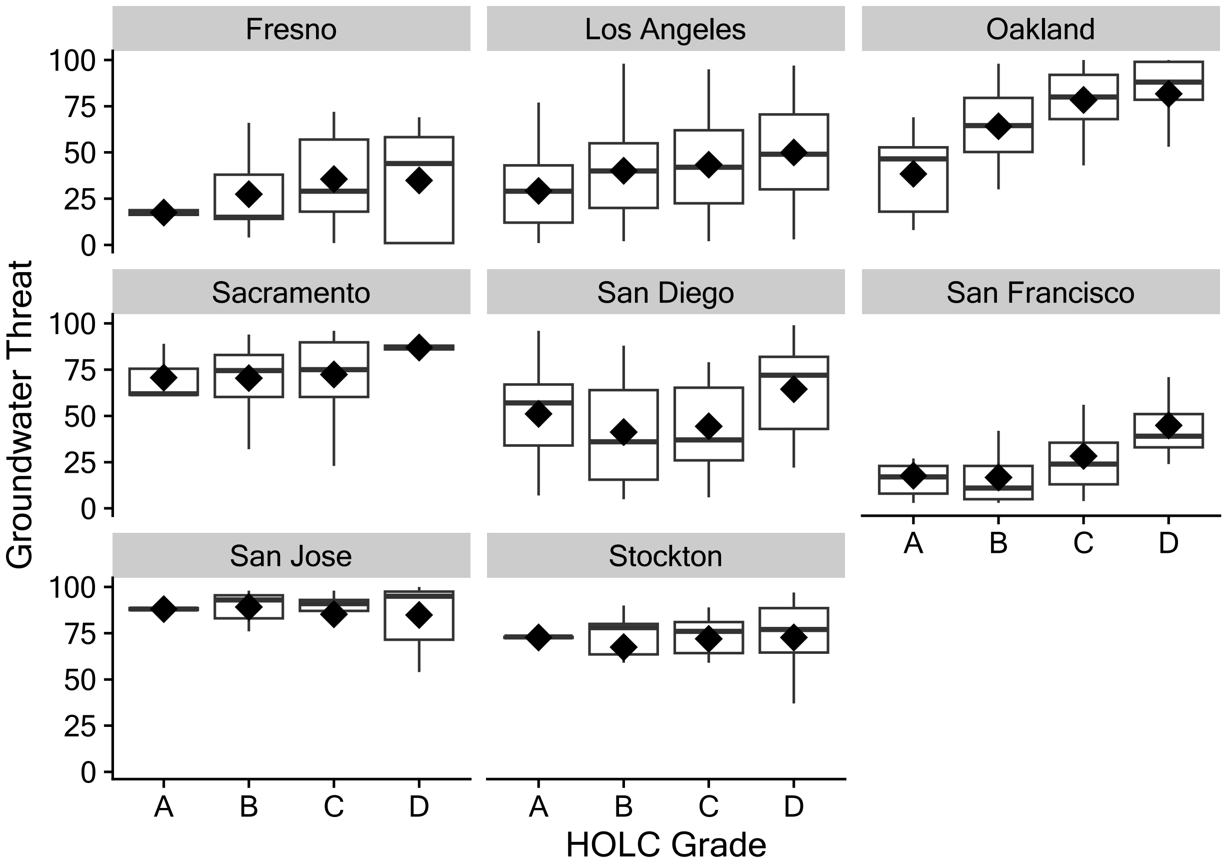

Figure S4. The relationship between HOLC grade and groundwater threat for each city in our dataset. Measurements are shown in box plots where each dot represents a measurement within a neighborhood. The mean is shown as a black diamond, and whiskers represent 95% confidence intervals.

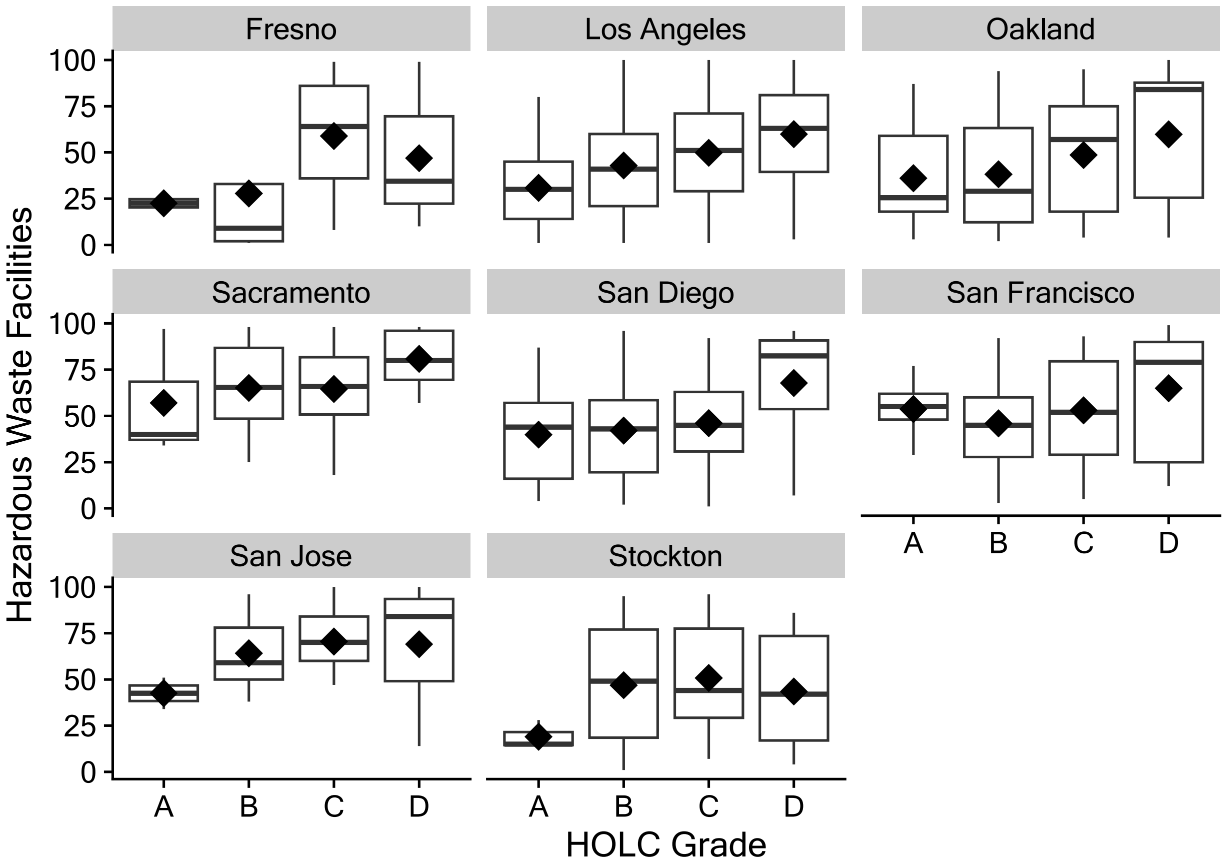

Figure S5. The relationship between HOLC grade and hazardous waste facilities for each city in our dataset. Measurements are shown in box plots where each dot represents a measurement within a neighborhood. The mean is shown as a black diamond, and whiskers represent 95% confidence intervals.

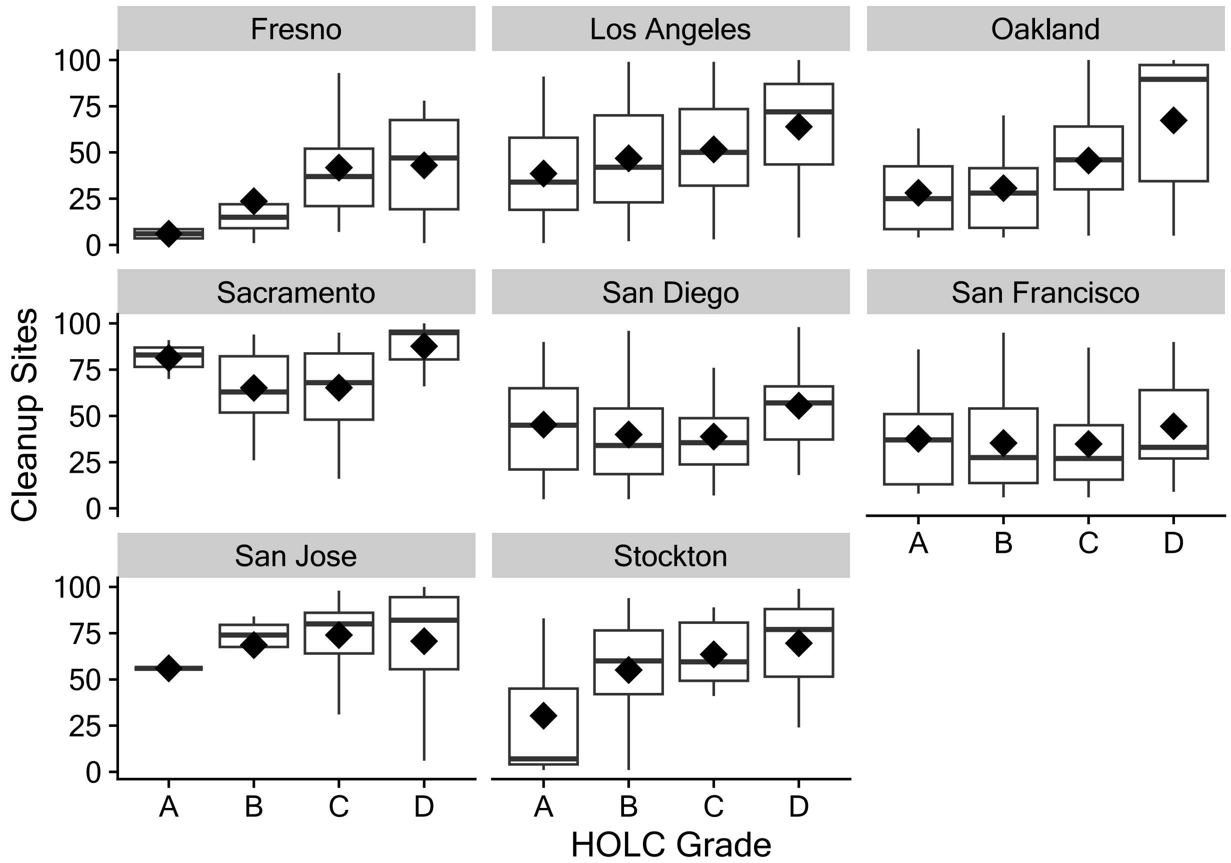

Figure S6. The relationship between HOLC grade and cleanup (i.e., brownfield) sites for each city in our dataset. Measurements are shown in box plots where each dot represents a measurement within a neighborhood. The mean is shown as a black diamond, and whiskers represent 95% confidence intervals.

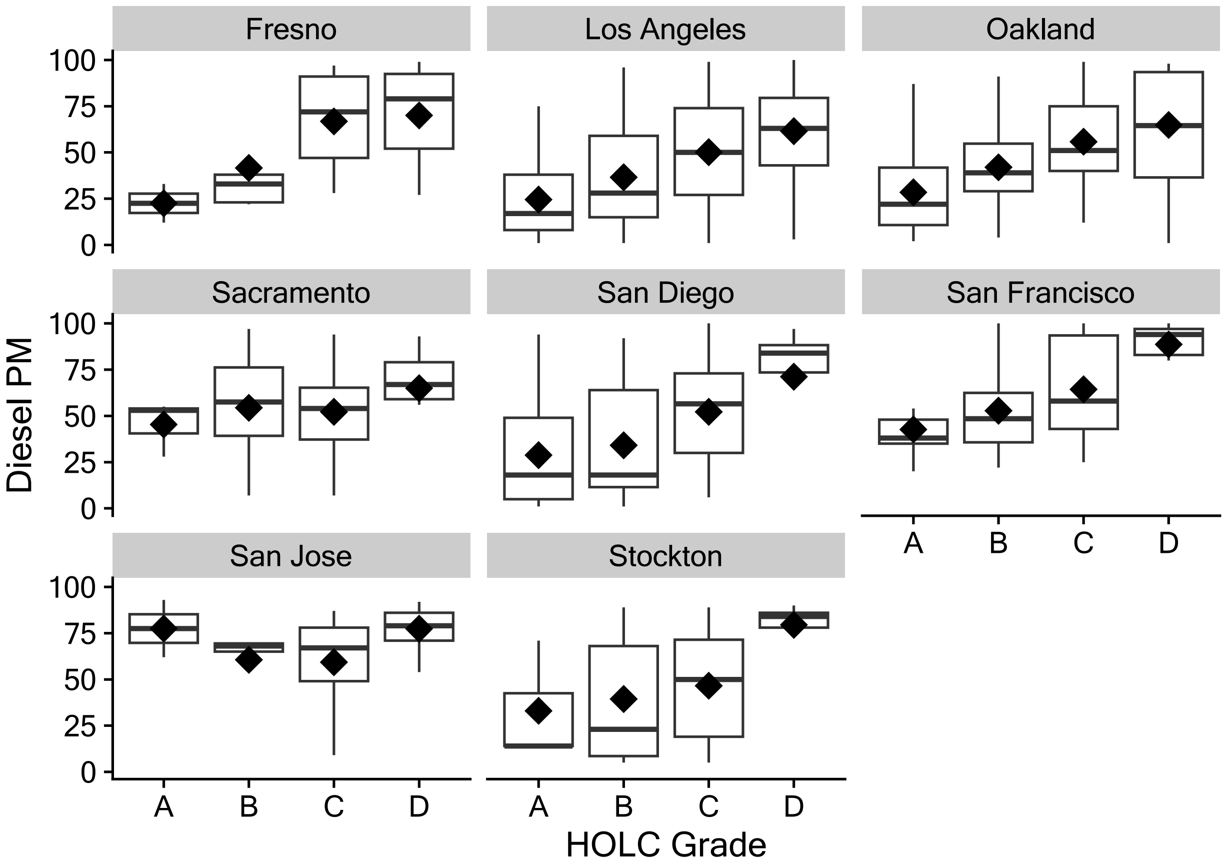

Figure S7. The relationship between HOLC grade and diesel particulate matter for each city in our dataset. Measurements are shown in box plots where each dot represents a measurement within a neighborhood. The mean is shown as a black diamond, and whiskers represent 95% confidence intervals.

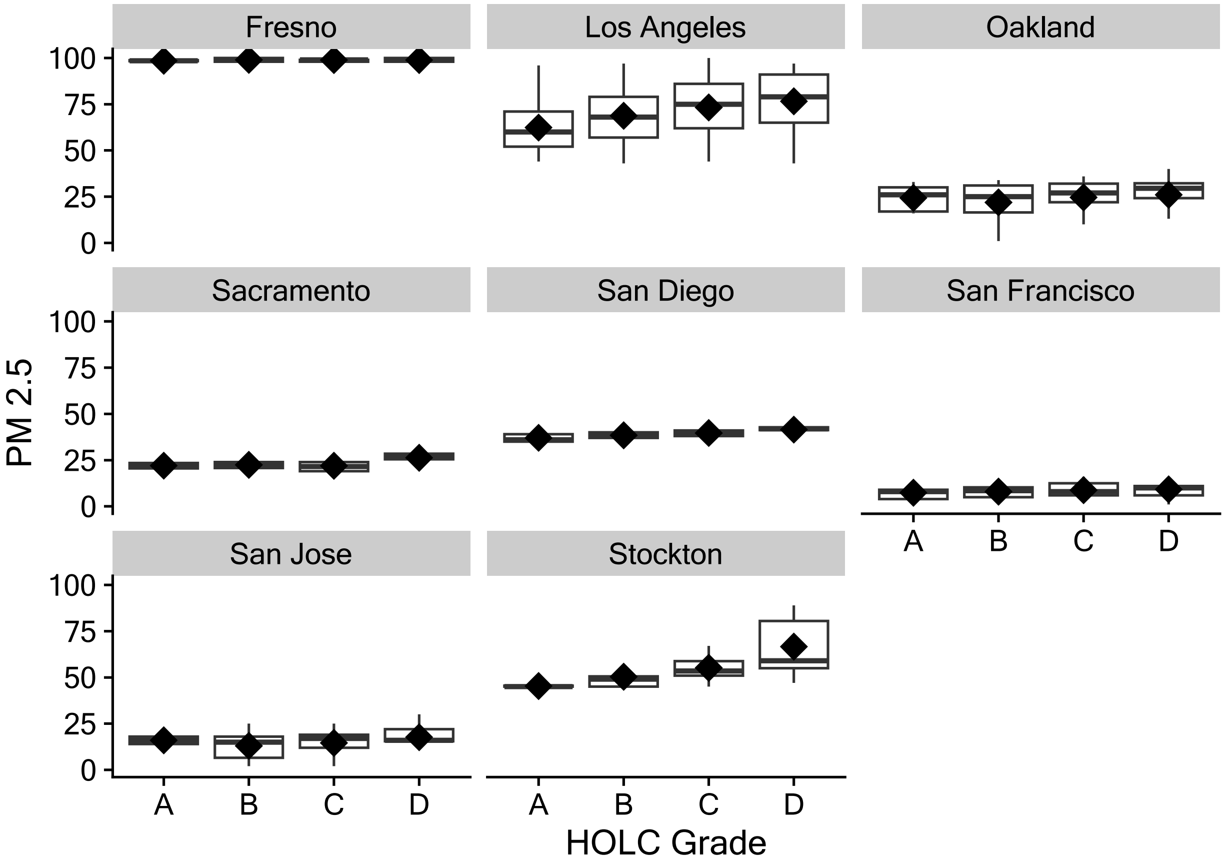

Figure S8. The relationship between HOLC grade and particulate matter 2.5 ($PM_{2.5}$) for each city in our dataset. Measurements are shown in box plots where each dot represents a measurement within a neighborhood. The mean is shown as a black diamond, and whiskers represent 95% confidence intervals.

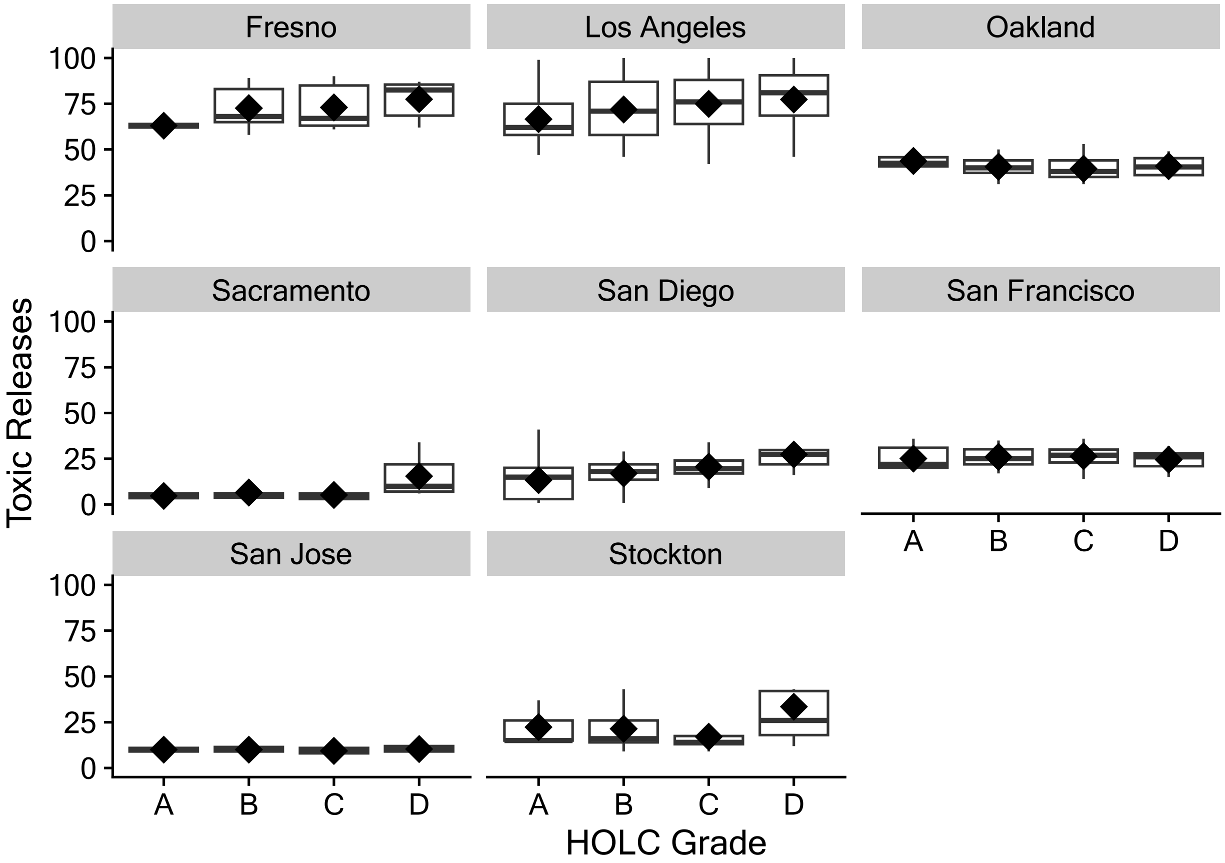

Figure S9. The relationship between HOLC grade and toxic releases from facilities for each city in our dataset. Measurements are shown in box plots where each dot represents a measurement within a neighborhood. The mean is shown as a black diamond, and whiskers represent 95% confidence intervals.

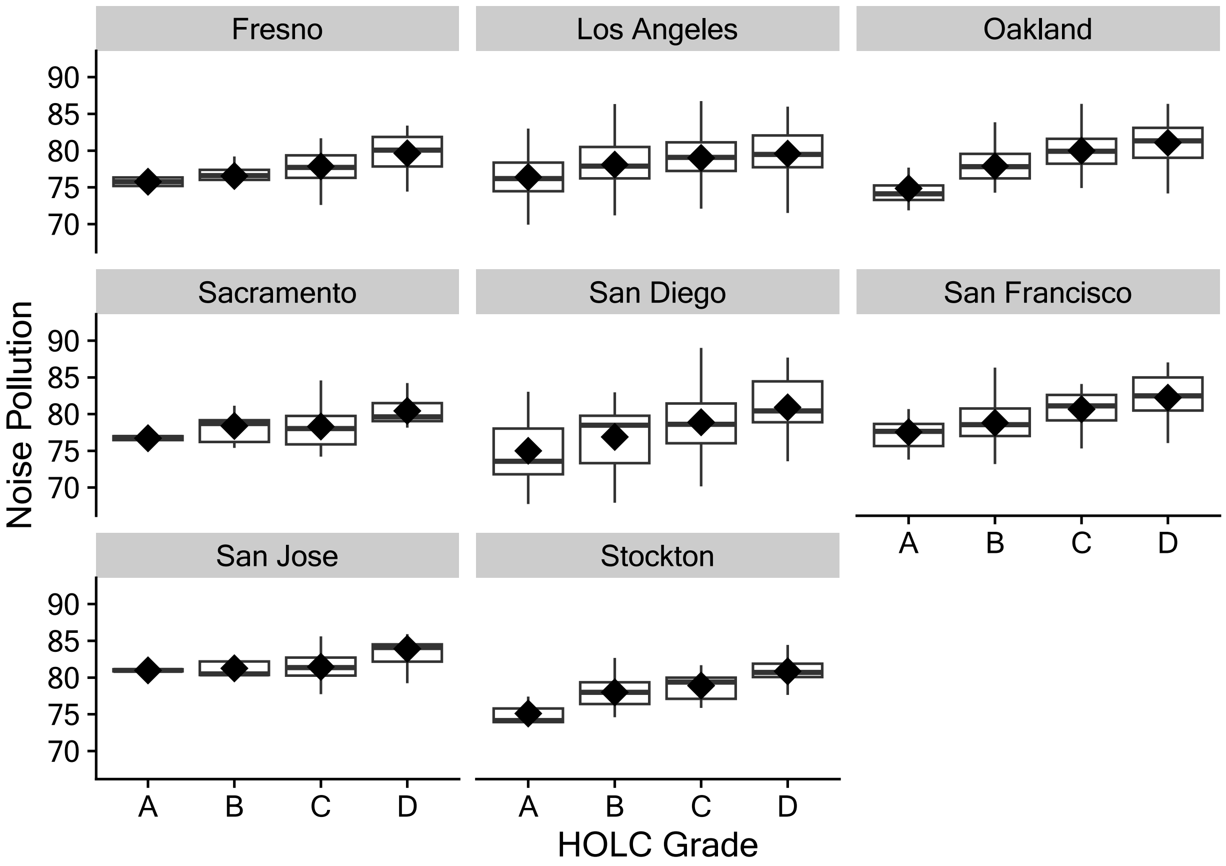

Figure S10. The relationship between HOLC grade and noise pollution for each city in our dataset. Measurements are shown in box plots where each dot represents a measurement within a neighborhood. The mean is shown as a black diamond, and whiskers represent 95% confidence intervals.

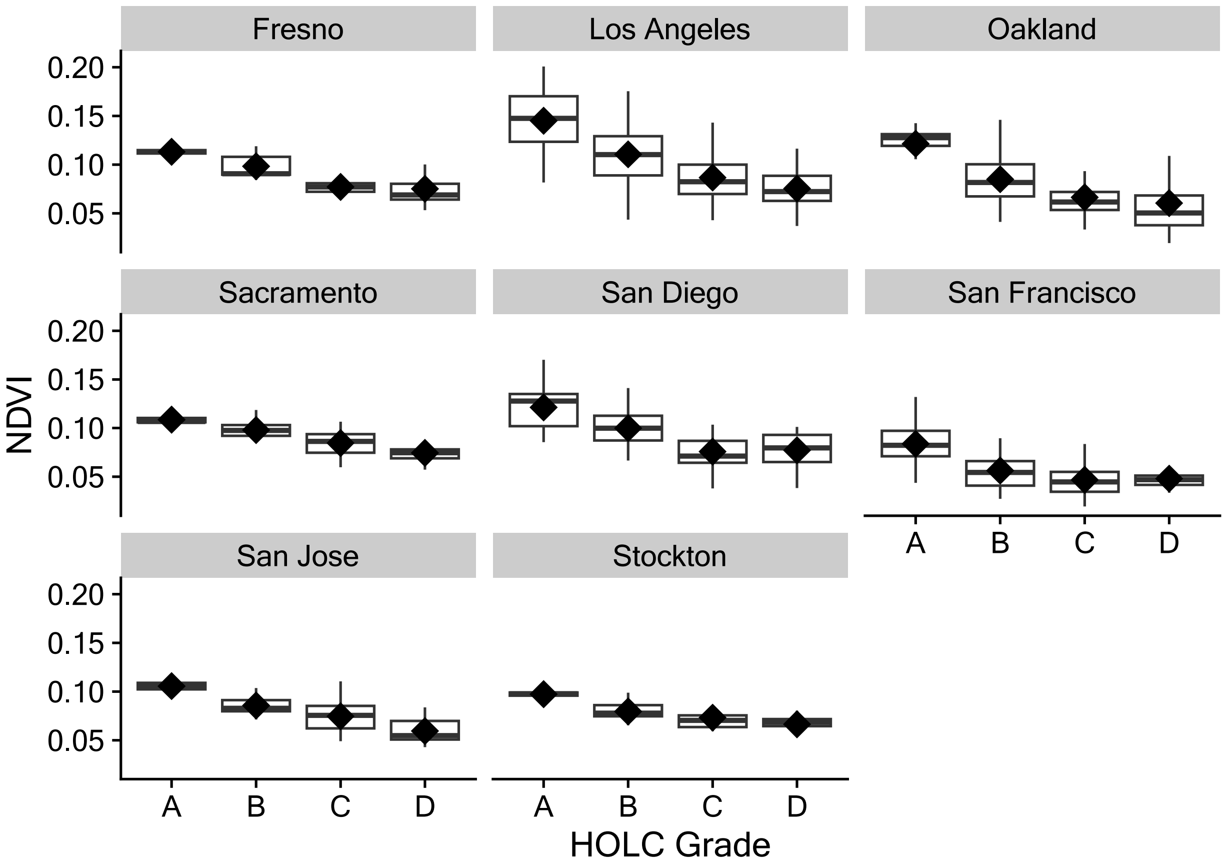

Figure S11. The relationship between HOLC grade and NDVI (i.e., vegetation) for each city in our dataset. Measurements are shown in box plots where each dot represents a measurement within a neighborhood. The mean is shown as a black diamond, and whiskers represent 95% confidence intervals.

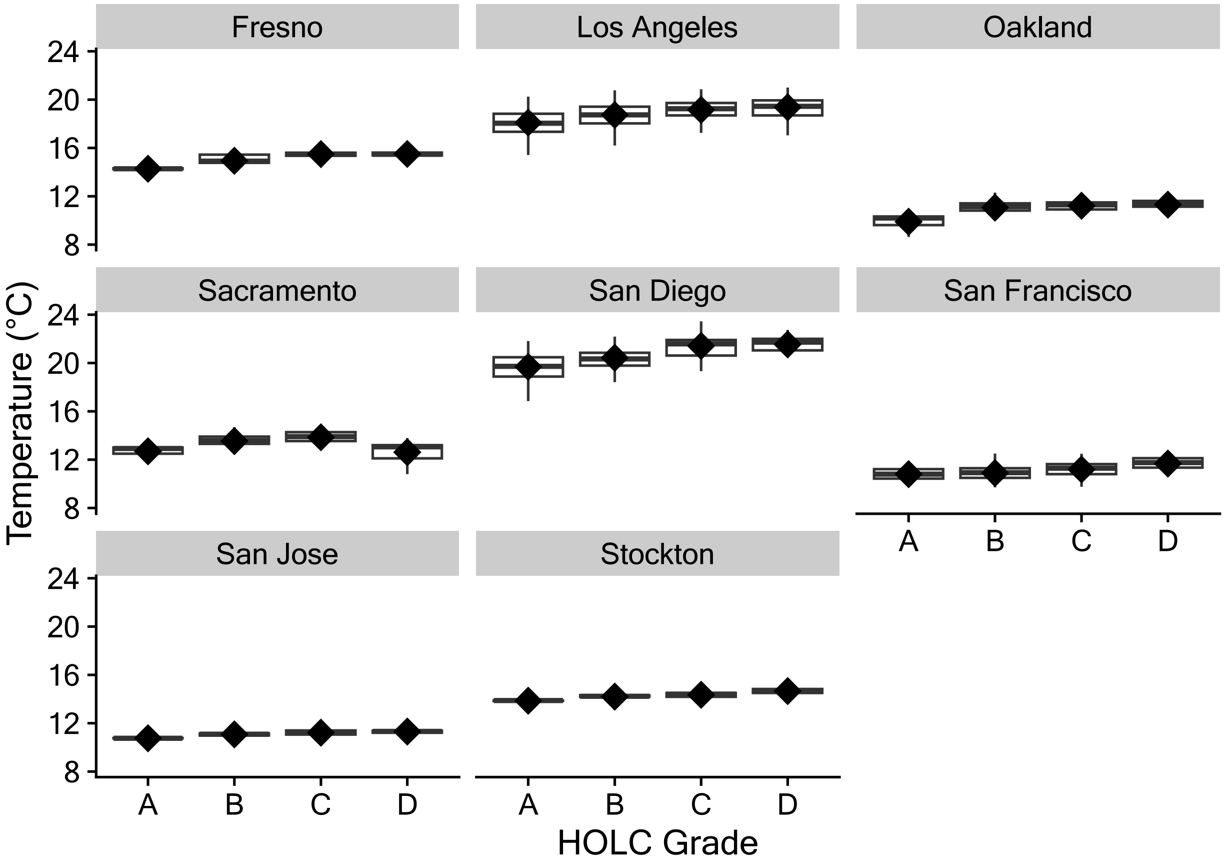

Figure S12. The relationship between HOLC grade and temperature for each city in our dataset. Measurements are shown in box plots where each dot represents a measurement within a neighborhood. The mean is shown as a black diamond, and whiskers represent 95% confidence intervals.

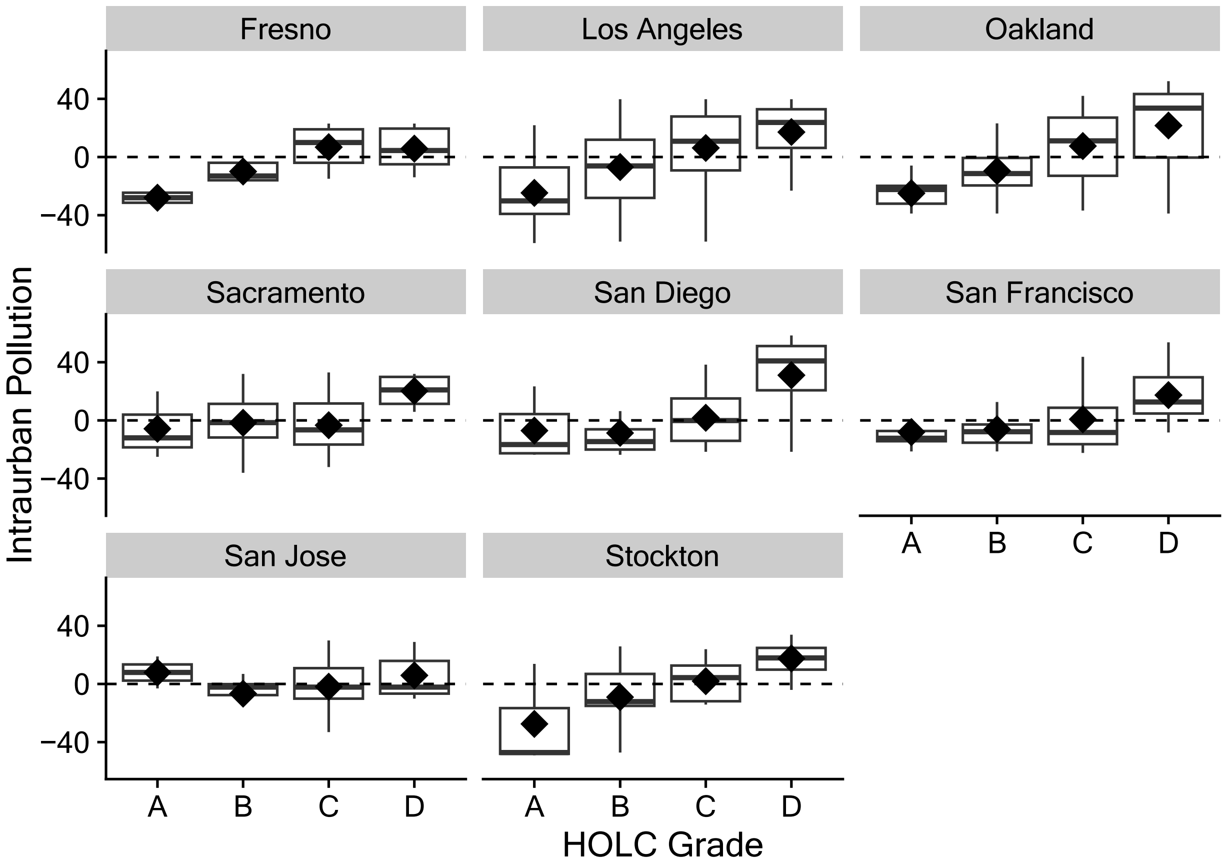

Figure S13. The relationship between HOLC grade and intraurban disparities in pollution burden for each city in our dataset. Measurements are shown in box plots where each dot represents a measurement within a neighborhood. The mean is shown as a black diamond, and whiskers represent 95% confidence intervals. Values above the line represent a higher pollution burden than the city's average.

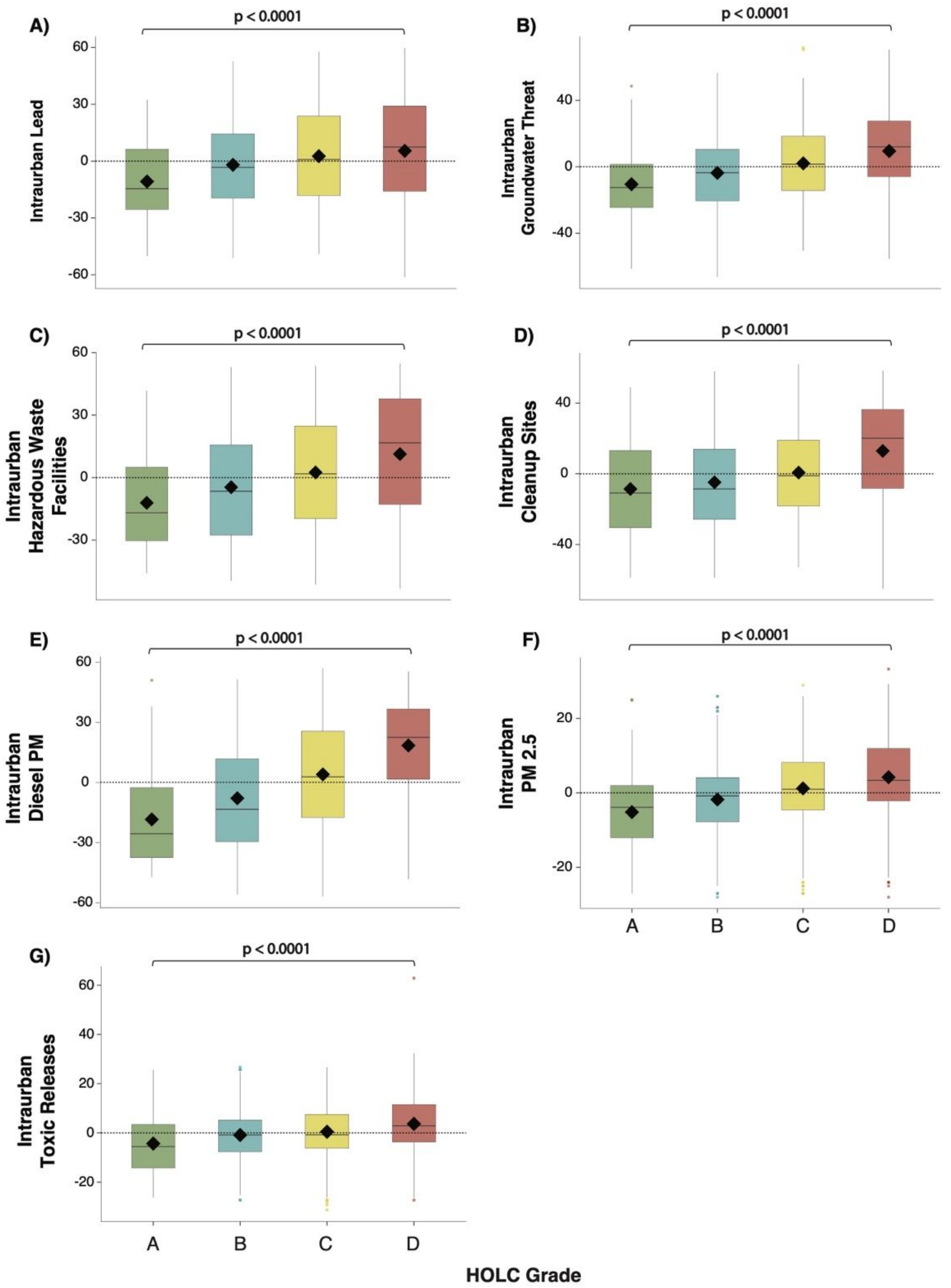

Figure S14. The relationship between HOLC grade and intraurban disparities for (A) lead risk from housing, (B) water contamination, (C) hazardous waste facilities, (D) cleanup sites, (E) diesel particulate matter, (F) particulate matter (pm) 2.5 and (G) toxic releases from facilities across formerly graded HOLC neighborhoods. The mean is shown as a black diamond, and whiskers represent 95% confidence intervals. Values above the line represent higher environmental hazard exposure than the city's average.

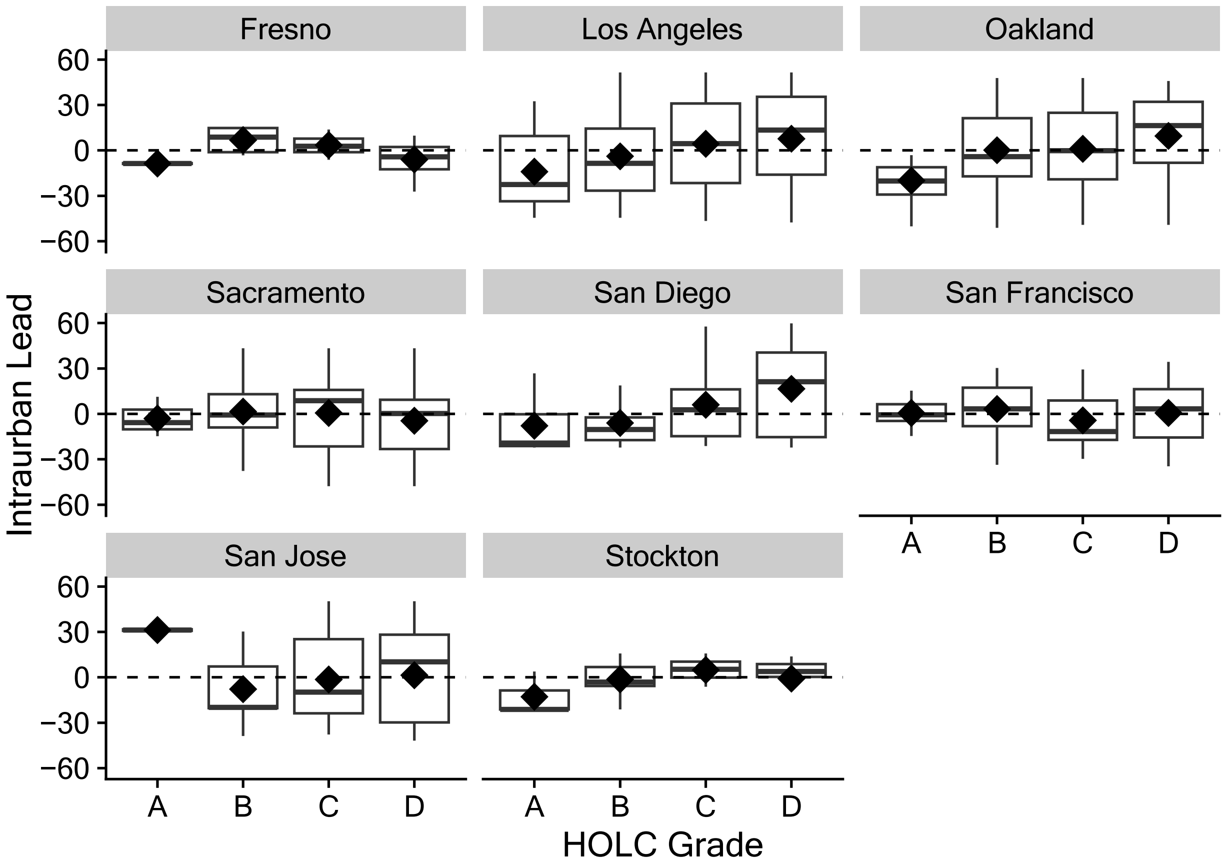

Figure S15. The relationship between HOLC grade and intraurban disparities in lead risk from housing for each city in our dataset. Measurements are shown in box plots where each dot represents a measurement within a neighborhood. The mean is shown as a black diamond, and whiskers represent 95% confidence intervals. Values above the line represent higher lead risk than the city's average.

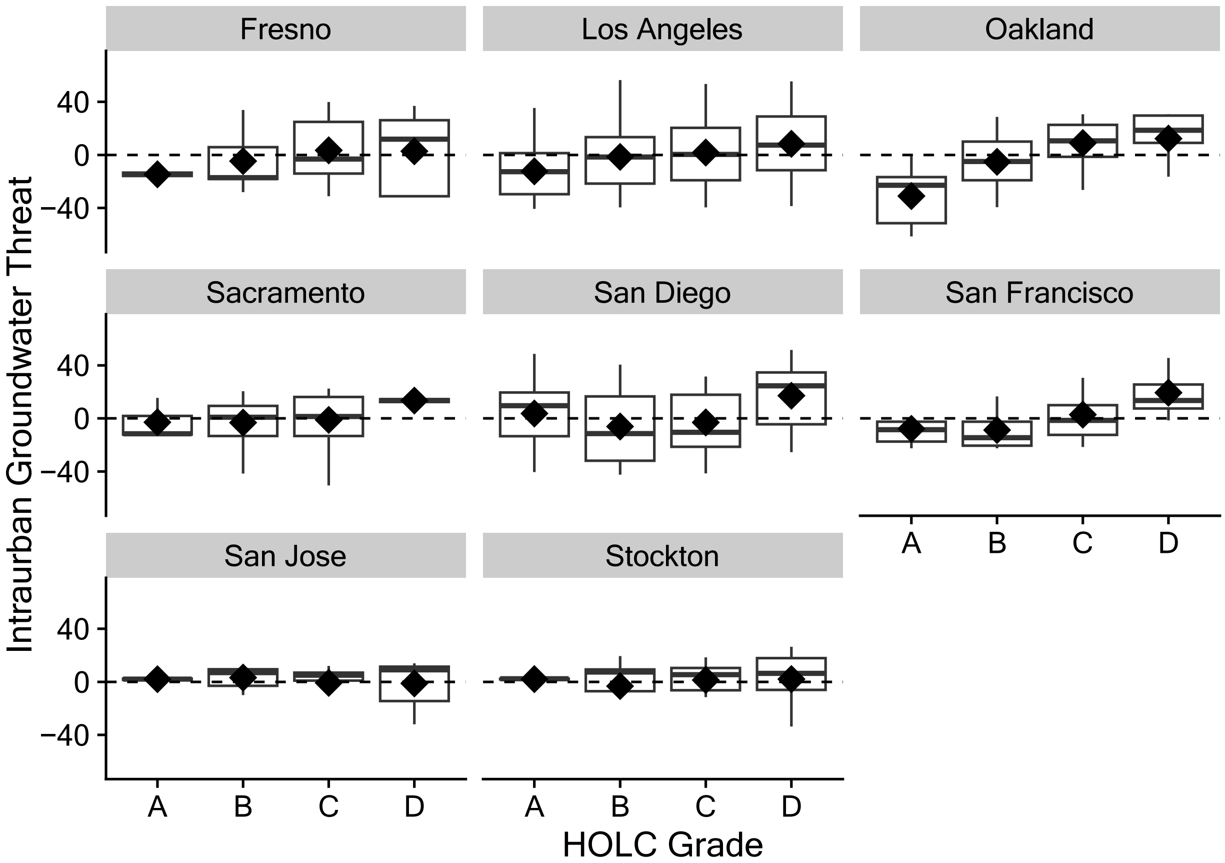

Figure S16. The relationship between HOLC grade and intraurban disparities in groundwater threat for each city in our dataset. Measurements are shown in box plots where each dot represents a measurement within a neighborhood. The mean is shown as a black diamond, and whiskers represent 95% confidence intervals. Values above the line represent higher levels of groundwater threat than the city's average.

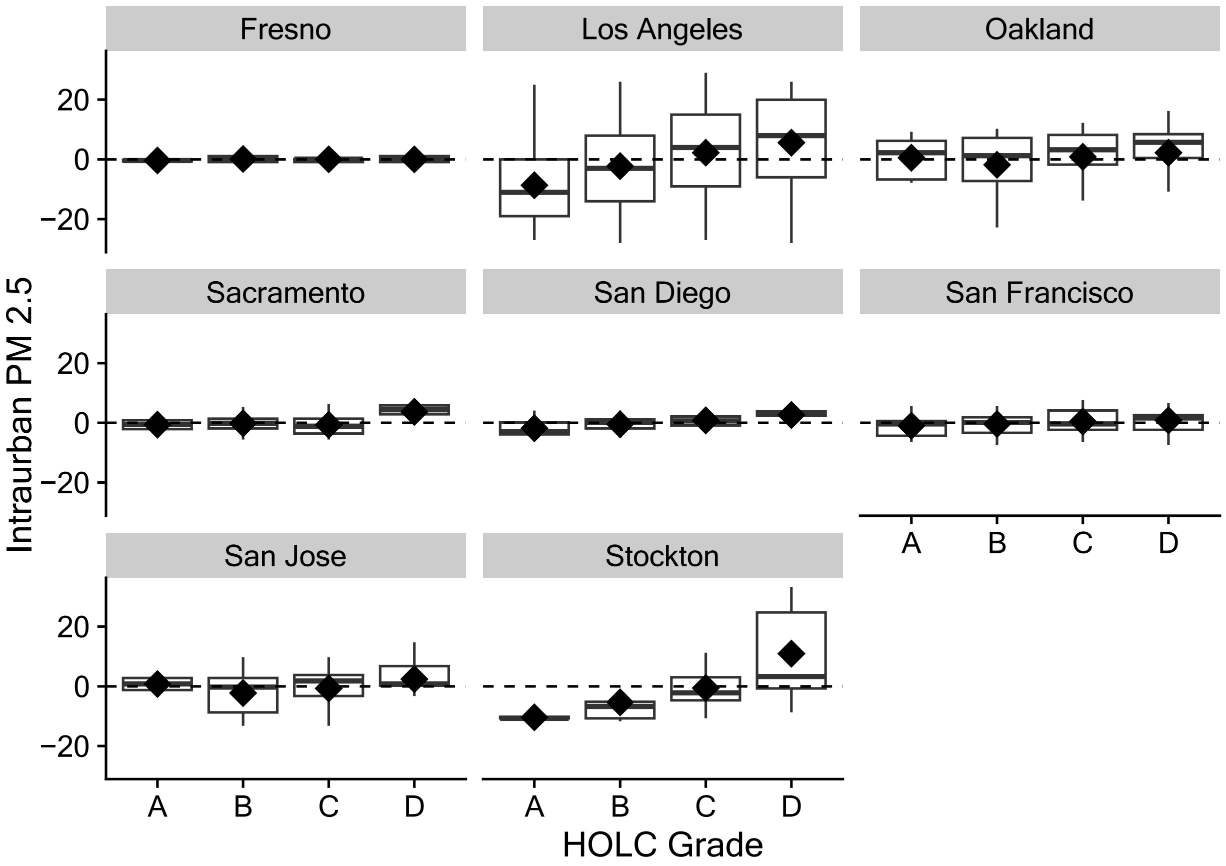

Figure S17. The relationship between HOLC grade and intraurban disparities in particulate matter 2.5 ($PM_{2.5}$) for each city in our dataset. Measurements are shown in box plots where each dot represents a measurement within a neighborhood. The mean is shown as a black diamond, and whiskers represent 95% confidence intervals. Values above the line represent higher levels of $PM_{2.5}$ than the city's average.

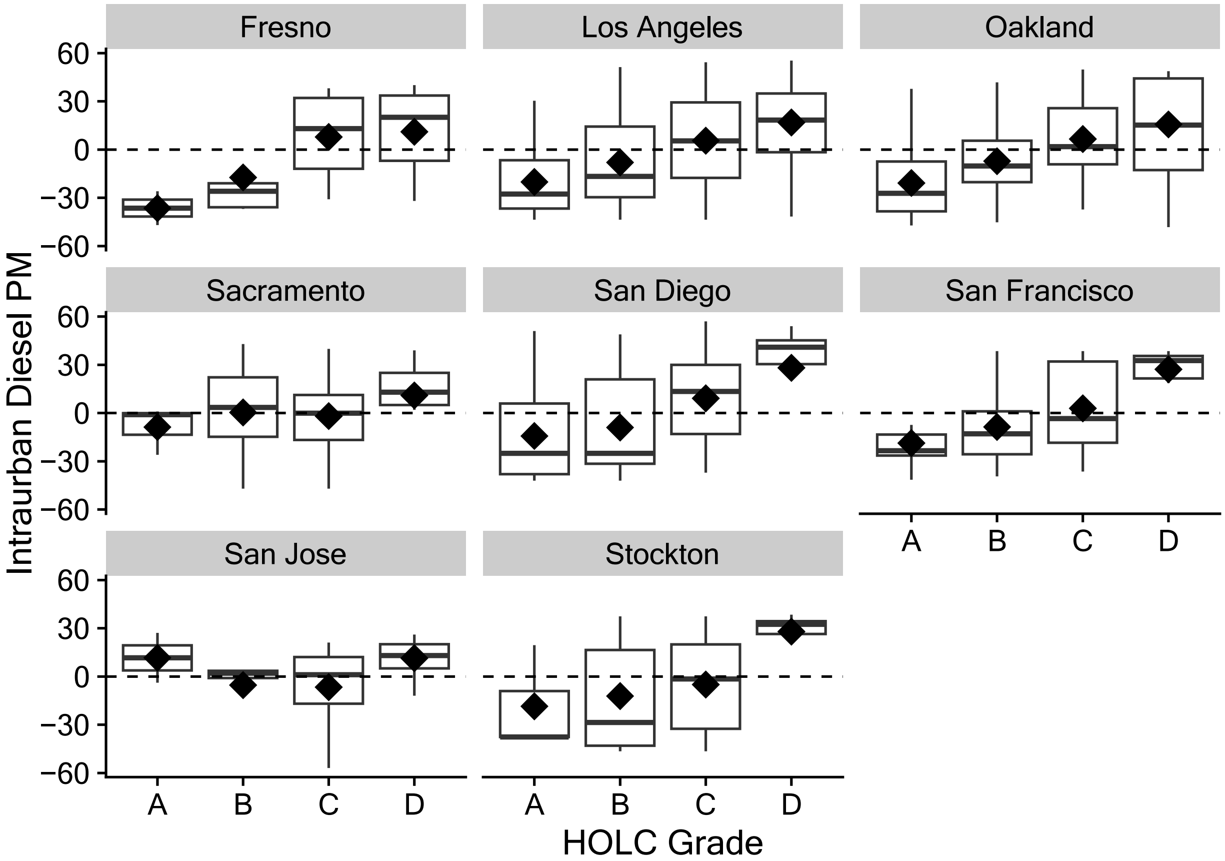

Figure S18. The relationship between HOLC grade and intraurban disparities in diesel particulate matter for each city in our dataset. Measurements are shown in box plots where each dot represents a measurement within a neighborhood. The mean is shown as a black diamond, and whiskers represent 95% confidence intervals. Values above the line represent higher levels of diesel particulate matter than the city's average.

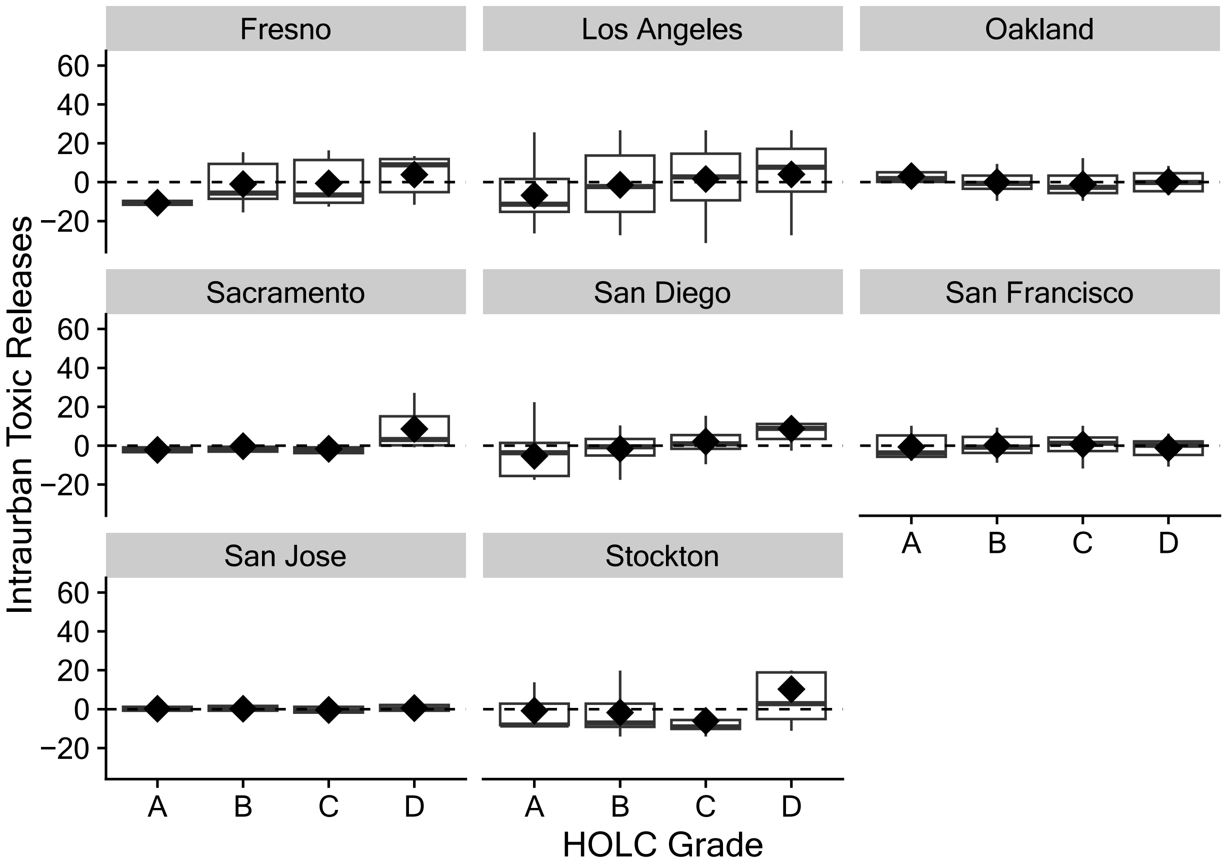

Figure S19. The relationship between HOLC grade and intraurban disparities in toxic releases from facilities for each city in our dataset. Measurements are shown in box plots where each dot represents a measurement within a neighborhood. The mean is shown as a black diamond, and whiskers represent 95% confidence intervals. Values above the line represent higher levels of toxic releases than the city's average.

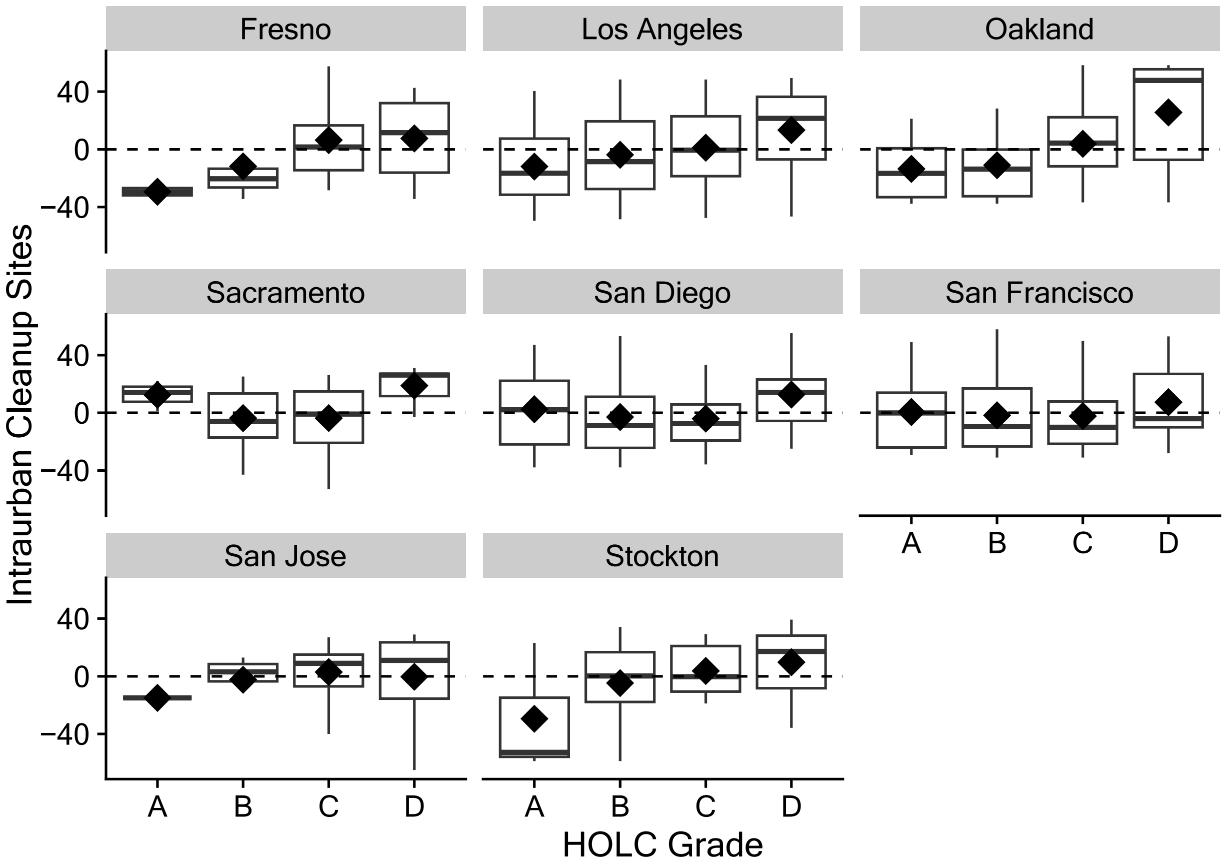

Figure S20. The relationship between HOLC grade and intraurban disparities in cleanup (i.e., brownfield) sites for each city in our dataset. Measurements are shown in box plots where each dot represents a measurement within a neighborhood. The mean is shown as a black diamond, and whiskers represent 95% confidence intervals. Values above the line represent higher amounts of cleanup sites than the city's average.

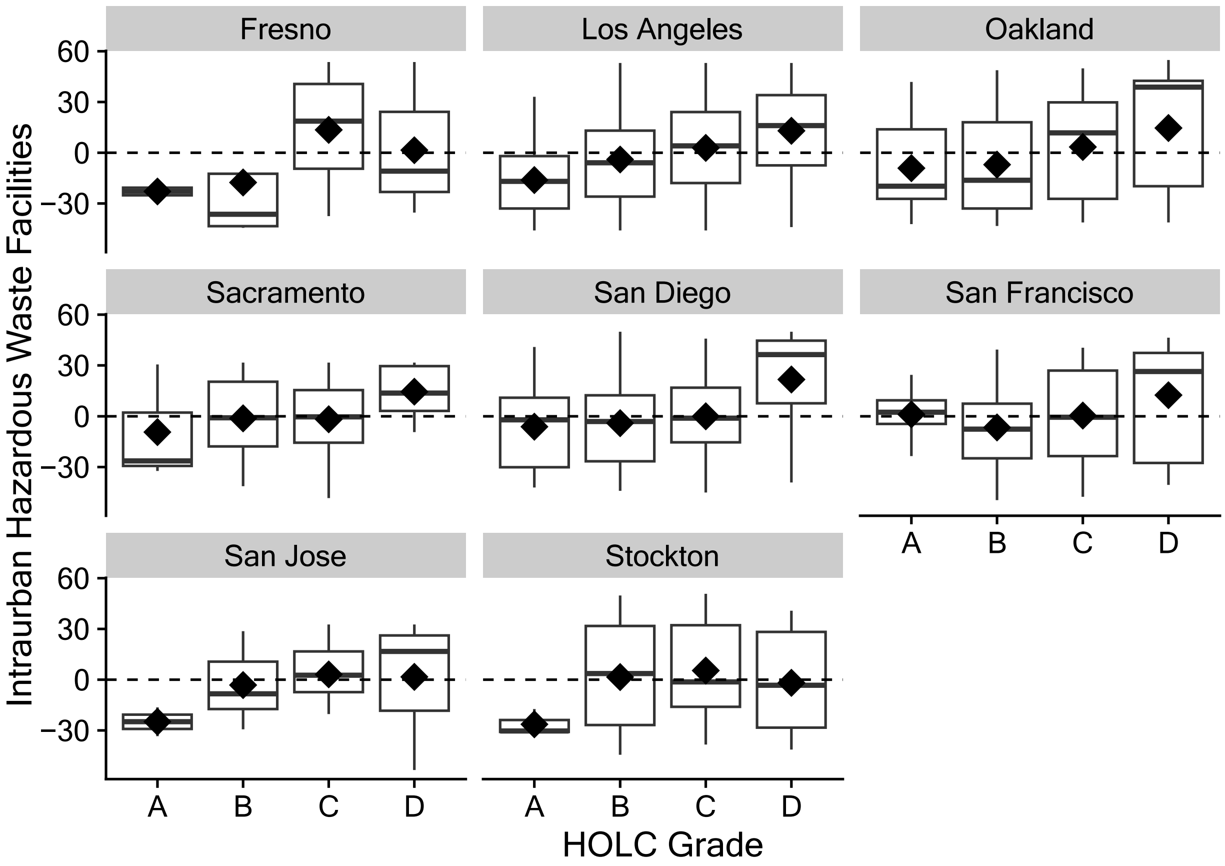

Figure S21. The relationship between HOLC grade and intraurban disparities in hazardous waste facilities for each city in our dataset. Measurements are shown in box plots where each dot represents a measurement within a neighborhood. The mean is shown as a black diamond, and whiskers represent 95% confidence intervals. Values above the line represent higher amounts of hazardous waste facilities than the city's average.

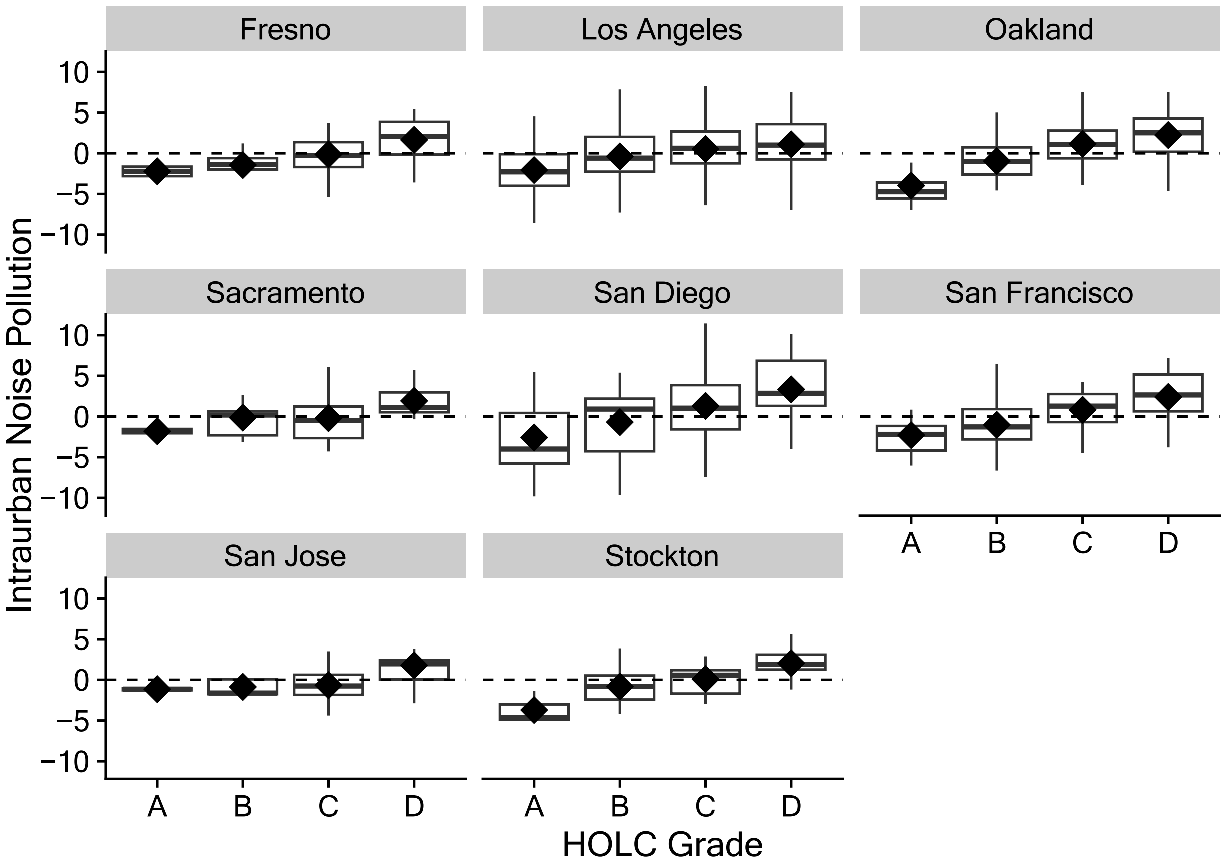

Figure S22. The relationship between HOLC grade and intraurban disparities in noise pollution for each city in our dataset. Measurements are shown in box plots where each dot represents a measurement within a neighborhood. The mean is shown as a black diamond, and whiskers represent 95% confidence intervals. Values above the line represent higher levels of noise pollution than the city's average.

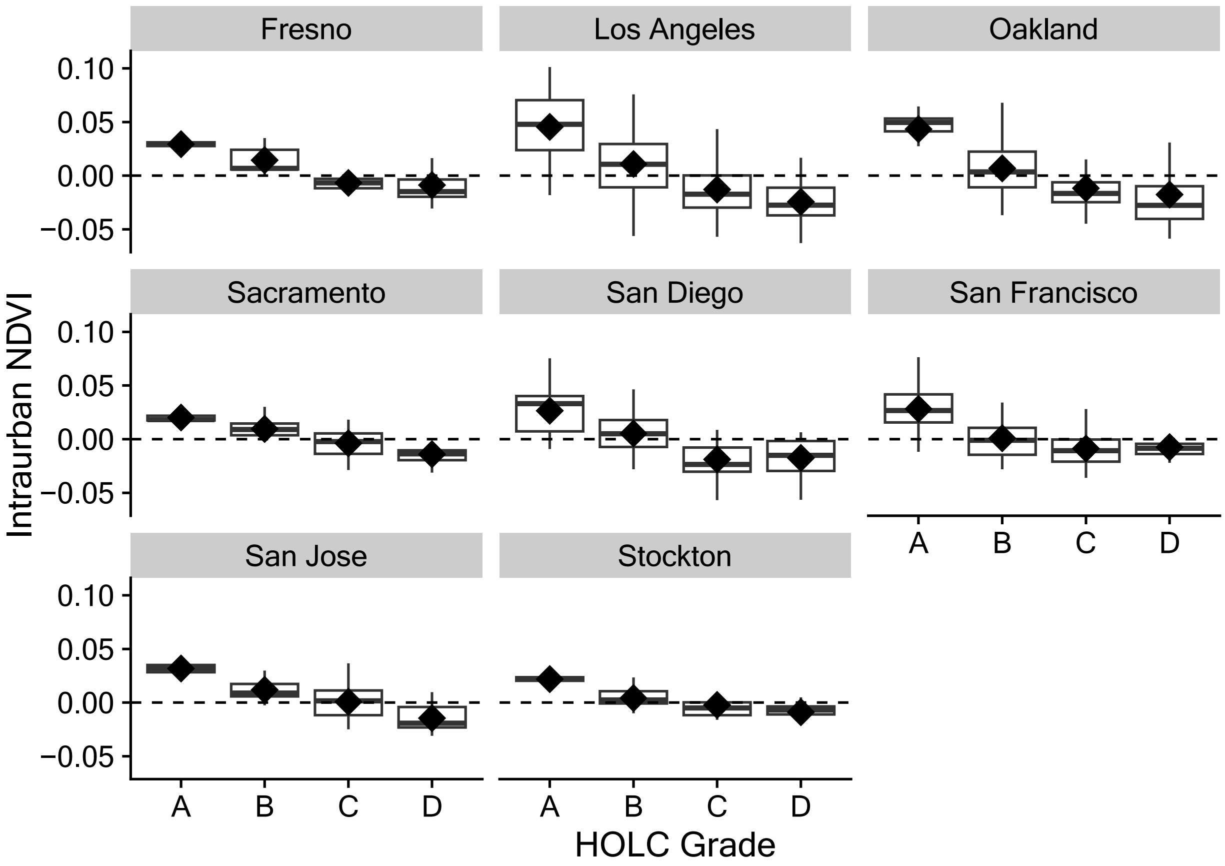

Figure S23. The relationship between HOLC grade and intraurban disparities in NDVI (i.e., vegetation) for each city in our dataset. Measurements are shown in box plots where each dot represents a measurement within a neighborhood. The mean is shown as a black diamond, and whiskers represent 95% confidence intervals. Values below the line represent less vegetation than the city's average.

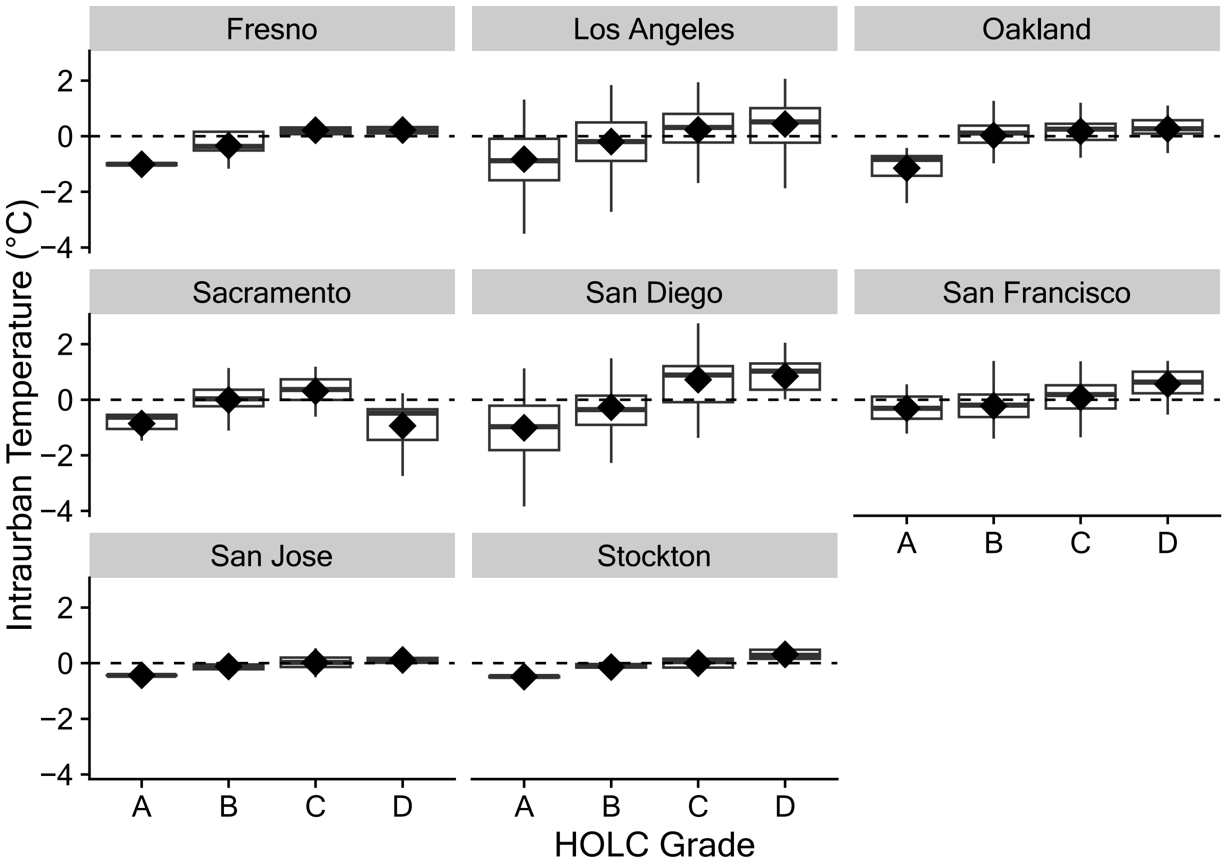

Figure S24. The relationship between HOLC grade and intraurban disparities in temperature for each city in our dataset. Measurements are shown in box plots where each dot represents a measurement within a neighborhood. The mean is shown as a black diamond, and whiskers represent 95% confidence intervals. Values above the line represent higher temperature than the city's average.
